# Single-molecule amplification-free multiplexed detection of circulating microRNA cancer biomarkers from serum

**DOI:** 10.1038/s41467-021-23497-y

**Published:** 2021-06-10

**Authors:** Shenglin Cai, Thomas Pataillot-Meakin, Akifumi Shibakawa, Ren Ren, Charlotte L. Bevan, Sylvain Ladame, Aleksandar P. Ivanov, Joshua B. Edel

**Affiliations:** 1 Department of Chemistry, Imperial College London, Molecular Science Research Hub, London, W12 0BZ UK; 2 Department of Bioengineering, Imperial College London, Sir Michael Uren Hub, London, W12 0BZ UK; 3 Department of Surgery and Cancer, Imperial College London, Hammersmith Hospital, London, UK

**Keywords:** Single-molecule biophysics, Tumour biomarkers, miRNAs, Nanobiotechnology

## Abstract

MicroRNAs (miRNAs) play essential roles in post-transcriptional gene expression and are also found freely circulating in bodily fluids such as blood. Dysregulated miRNA signatures have been associated with many diseases including cancer, and miRNA profiling from liquid biopsies offers a promising strategy for cancer diagnosis, prognosis and monitoring. Here, we develop size-encoded molecular probes that can be used for simultaneous electro-optical nanopore sensing of miRNAs, allowing for ultrasensitive, sequence-specific and multiplexed detection directly in unprocessed human serum, in sample volumes as small as 0.1 μl. We show that this approach allows for femtomolar sensitivity and single-base mismatch selectivity. We demonstrate the ability to simultaneously monitor miRNAs (miR-141-3p and miR-375-3p) from prostate cancer patients with active disease and in remission. This technology can pave the way for next generation of minimally invasive diagnostic and companion diagnostic tests for cancer.

## Abstract (web summary)

miRNA profiling from patient blood can be used for cancer diagnosis. Here the authors present an electro-optical nanopore sensing platform which allows sensitive and specific miRNA detection directly in human serum and apply to monitoring of miR-141-3p and miR-375-3p in different stage of prostate cancer.

## Introduction

Prostate cancer (PCa), diagnosed at a mean age of 66 years^1^, is the second most prevalent male cancer representing 13.5% of all cases and 6.7% of all male cancer-related deaths globally^2^. Current monitoring of PCa relies heavily on the detection of serum prostate-specific antigen (PSA), also used in diagnosis. However, these blood tests have low specificity and sensitivity, and refinements in methodology and interpretation have yielded only marginal improvements in accuracy^3–5^. PSA levels are subject to transient variations, and there is a significant lag time between PSA response and treatment effect or relapse, requiring confirmatory, clinically detectable changes in tumour volume in order to alter treatment regimens^6,7^. As a result, PCa is commonly associated with a high rate of overdiagnosis and overtreatment and reliance on PSA for monitoring is potentially causing clinicians to miss optimal therapeutic windows^5^.

Liquid biopsies, primarily blood and also urine, seminal or spinal fluids, represent a valuable source of biomarkers such as circulating cells, exosomes and nucleic acids^8,9^. These may give a more holistic and granular view of PCa than PSA, potentially allowing for a more rapid clinical response^10^. However, liquid biopsy candidates such as the genetic markers of PCA3 expression have not, as yet, shown greater efficacy than PSA for assessing disease progression or outcomes^11^. This is at least in part due to the lack of suitable analytical technologies for the accurate detection of such scarce molecular biomarkers directly from bodily fluids.

MicroRNAs (miRNAs) are short (18–22 nucleotides) non-coding RNAs involved in post-transcriptional repression of mRNA and found dysregulated in multiple cancer types^9,12,13^. MiR-141-3p and miR-375-3p in particular, are significantly overexpressed in the blood of PCa patients (compared to healthy individuals) and are implicated in several processes affecting tumorigenesis and metastasis^14,15^. Their potential for diagnosis and monitoring of PCa has been proposed, either as individual biomarkers or in combination^13,15–17^. Despite the potential of these and other miRNAs as minimally invasive biomarkers, none thus far have been translated into the clinic. It has been suggested that limitations of conventional technologies such as RNA extraction (labour/resource-intensive, preventing individual investigation of the various compartments in which circulatory miRNAs are found) or PCR amplification (further predisposing to pre-analytical inconsistencies, amplification bias and restricting quantification to relative terms) are major factors in this lack of translation^18,19^.

Many upcoming technologies are focused on addressing and eliminating these issues via amplification- and/or extraction-free methodologies with a push towards more point-of-care testing. Techniques including bead- or electrode-bound capture probes, and microfluidics, drive many of these advanced approaches; however, they often require expensive consumables or large sample volumes^20^. Single-molecule methods, such as fluorescence microscopy, can of course also be used for the detection of miRNAs.^21,22^ However, the complexity dramatically increases as the number of biomarkers being detected is also increased. There will be a limit to how many fluorophores can be used, especially at the single-molecule level. Label-free methods, such as nanopore sensors have also recently emerged as a versatile tool, in particular for performing measurements with single-molecule sensitivity^23–28^ including the detection of miRNAs^29–32^. However, to date, these approaches have been generally limited in their multiplexing capacity and lack the sensitivity and selectivity required for accurate and specific detection of clinically relevant but rare and highly homologous species.

Here, we showcase an electro-optical sensing platform combining the benefits of single-molecule nanopore sensing with fluorescence microscopy for use in label-free, unamplified detection of miRNAs directly in patient samples. This is achieved by designing custom molecular probes consisting of a DNA carrier and a molecular beacon (MB) that can be used to target and attract individual miRNAs selectively. In our study, irrespective of the number of biomarkers, only a single fluorophore is needed simplifying the overall complexity of the experiment. We show that this approach allows for femtomolar sensitivity and single-base mismatch selectivity. The capability of our platform was validated using only 0.1 μl of unextracted patient serum samples to quantify the concentration of miR-141-3p and miR-375-3p in PCa patients with active disease compared to those in remission. We also show the mapping of 3 miRNAs to classify different PCa stages (i.e. remission, localised and metastatic). Our results demonstrate that this is a highly sensitive and amplification-free method of quantifying circulating miRNAs in blood serum and can potentially be used as a clinical monitoring tool, with applications not only for PCa but also for a wide range of cancers and other diseases.

## Results

### Multiplexed electro-optical nanopore sensing

**Fig. 1:**
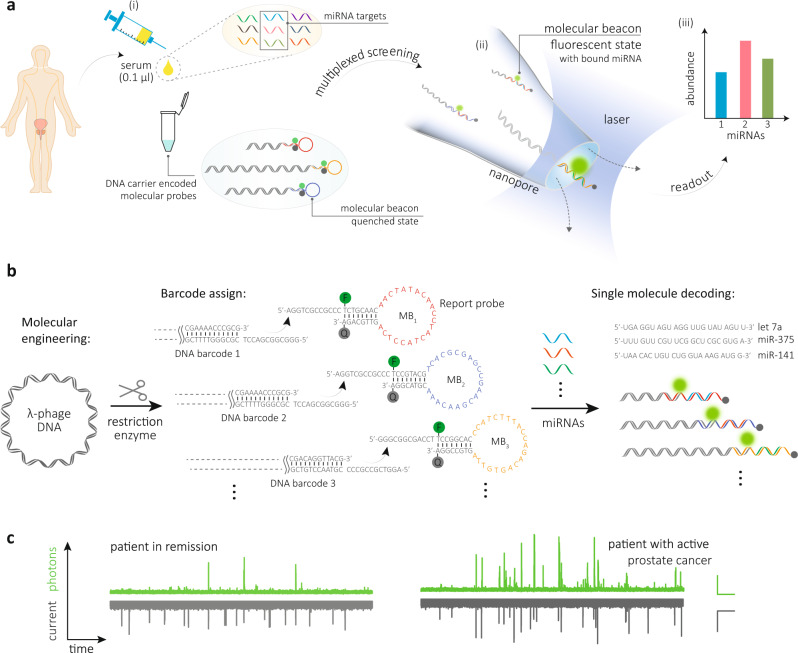
Single-molecule multiplexed sensing using an electro-optical nanopore platform. **a** Workflow for detection of miRNAs directly from patient serum. (i) Serum from patients was incubated with length-encoded molecular probes consisting of a DNA carrier and molecular beacon (MB). (ii) Electro-optical sensing was then performed, and (iii) the miRNA expression levels were determined. **b** Schematic representation of the preparation of size-coded DNA probes and their binding to respective miRNA targets. Lambda-phage DNA is enzymatically engineered to generate different size fragments with 12-base single-stranded termini. Customised MB sequences are then assigned and hybridised to these fragments. Upon binding to the corresponding miRNAs, the ‘stem-loop’ structure unfolds, resulting in an increase in fluorescence intensity induced by the increase in distance between the fluorophore and the quencher. **c** Representative photon and current time traces for simultaneous detection of miR-141-3p and miR-375-3p from a patient in remission and a patient with active prostate cancer. Traces were recorded using asymmetric KCl buffer conditions (inside/outside nanopipette, 40/400 mM) at −300 mV bias and a laser power of 90 ± 4 µW. Scale bar of photon trace (green): vertical, 100 counts/ms, horizontal, 2.5 s. Scale bar of current trace: vertical, 30 pA, horizontal, 2.5 s.

The electro-optical nanopore sensing workflow and platform involved the alignment of a nanopipette tip with a diffraction-limited confocal detection volume (Fig. 1a), as we recently reported^33^. Details of the experimental set-up, characterisation of nanopipettes and the alignment protocol can be found in the ‘Methods’ section and Supplementary Figs. 1–4. To selectively sense short oligonucleotides, such as miRNA, a molecular probe was designed consisting of an MB coupled to a double-stranded (dsDNA) carrier >5 kilobase pair (kbp) in length. The MB is a stem-loop structured oligonucleotide, labelled with a fluorophore and a quencher. Upon sequence-specific hybridisation to a complementary miRNA, the stem-loop opens, thus spatially separating the quencher from the fluorophore and restoring its intrinsic emissive properties, Fig. 1b. The optical signal, therefore, gives a binary yes/no response confirming whether a miRNA is bound to the MB. Meanwhile, the nanopore electrical signal depends on the length of the dsDNA carrier and acts as an internal barcode to confirm which miRNA is being detected. For multiplexed detection, DNA carriers of different lengths are barcoded into MB probes to produce unique transients in the ionic current, Fig. 1b. For example, the dwell time (∆*t*) and the peak current area (the integrated current over event dwell time) are proportional to the size of the DNA carriers. The presence of a specific miRNA can be confirmed by observing synchronised events in the optical and electrical detection channels, Fig. 1c. Since only a fraction of miRNA will bind to the probes and produce an optical signal, the concentration of miRNA can thus be quantified by determining the fraction of synchronised events relative to the total number of events in the electrical channel^33^.

### Design and characterisation of DNA carrier-encoded molecular probes

**Fig. 2:**
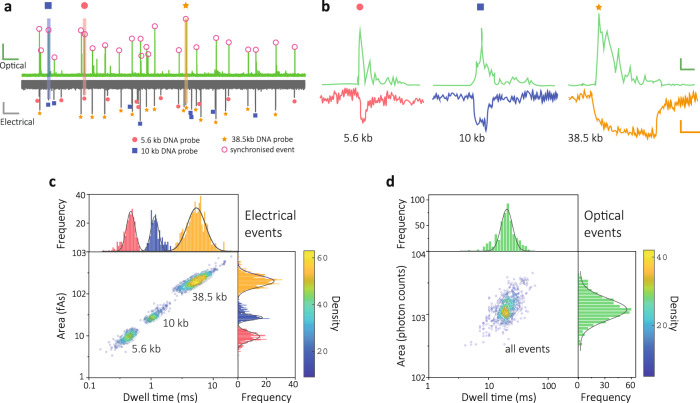
Length-encoded molecular probes consisting of DNA carriers and molecular beacons. **a** Representative photon and current time traces for translocation of 5.6, 10 and 38.5 kbp DNA carrier-encoded molecular probes (10 pM each) in the presence of 10× of target miRNAs (let-7a, miR-375-3p and miR-141-3p). Three electrical signals representing the translocation events of 5.6, 10 and 38.5 kbp DNA fragment lengths are marked with a filled circle, square and asterisk. Signals in the optical channel with corresponding synchronised events are marked with circles. Scale bar of photon trace (green): vertical, 100 counts/ms, horizontal, 2.5 s. Scale bar of current trace: vertical, 20 pA, horizontal, 2.5 s. **b** Zoom-in views for the three respective classes of events for size codes of 5.6, 10 and 38.5 kbp molecular probes. Scale bar of photon trace: vertical, 50 counts/ms, horizontal, 10 ms. Scale bar of current trace: vertical, 10 pA, horizontal, 1 ms. **c**, **d** Density scatter plots of dwell time vs peak current area for the electrical (*n* = 1014) and optical (*n* = 507) signals. Three populations can be observed for the electrical events that represent the three DNA fragments, while only one population is observed for optical events. Histograms are fit using a Gaussian function. All the translocations were performed in 100 mM KCl buffer (5 mM MgCl_2_, 10 mM Tris-HCl, 1 mM EDTA, pH = 8.0) at an applied potential bias of −300 mV with laser power of 90 ± 4 µW.

To synthesise the DNA-encoded molecular probes, a two-step strategy was used, Fig. 1b. Firstly, enzymatically digested fragments of 48.5 kbp lambda-phage DNA were used as the DNA carrier, in part due to the ease of detection and because they offer 12-base sticky overhangs that can be used to bind to the MBs^33,34^. DNA fragments of three unique sizes were used, with lengths of 10 and 38.5 kbp generated via Apa I digestion, and 5.6 kbp generated via BstE II digestion. Secondly, MBs with customised sequences were grafted onto the sticky ends of these fragments using standard hybridisation protocols. Details of the design, preparation protocols and gel characterisation are provided in ‘Methods’ and Supplementary Figs. 5 and 6. Nanopore experiments were performed to electrically discriminate these DNA carriers, which is a requirement for multiplexed miRNA detection. Translocation experiments were performed and electro-optical traces were recorded (at a concentration of 10 pM of each DNA carrier with miRNA bound at 1:10 ratio) in a 100 mM KCl buffer (5 mM MgCl_2_, 10 mM Tris-HCl, 1 mM EDTA, pH = 8.0) at a voltage of −300 mV, Fig. 2a. Three distinct current transients were observed, with dwell times (∆*t*) of 0.45 ± 0.19, 1.1 ± 0.3, and 5.0 ± 4.1 ms (mean ± s.d.) and peak current areas (*A*) of 9.3 ± 5.5, 26.9 ± 7.1, and 189.7 ± 123.5 fAs (mean ± s.d.), for the 5.6, 10, and 38.5 kbp fragments, respectively, Fig. 2b, c. The optical events only consisted of a single population, Fig. 2b, d. It should be noted that folded states of the DNA carrier for a small fraction of events can be observed; however, this does not affect the performance of a co-incident analysis. Taking these results together, the combination of dwell time and peak current area of electrical recordings can be employed to discriminate different length-encoded molecular probes.

### Single-molecule multiplexed miRNA detection

Validation and testing of the molecular probes were initially performed for the detection of miR-375-3p and miR-141-3p. These two miRNA targets were chosen due to published evidence of their upregulation in PCa patients^16,35^. Furthermore, let-7a^36^ and miR-21^37^ were also selected due to their implication in multiple tumour types and frequent investigation as biomarkers in many cancers, such as breast, ovarian and colorectal cancer^38–40^. MB sequences complementary to miR-375-3p and miR-141-3p were attached to the 10 and 38.5 kbp DNA carriers through sequence-specific hybridisation and were denoted as Probe-375 (10 kb) and Probe-141 (38.5 kb), respectively. Measurements were performed at molecular probe concentrations of 10 pM.

**Fig. 3:**
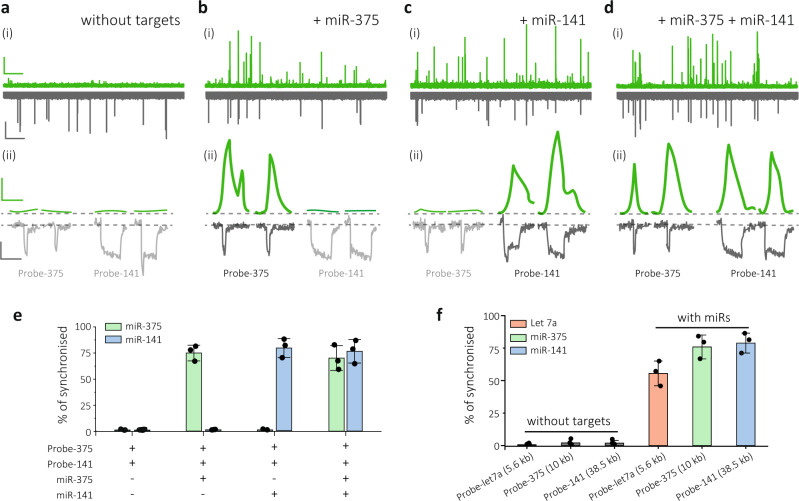
Single-molecule multiplexed detection using synthetic miRNAs. **a**–**d** Intensities-time traces (i) and representative events (ii) for the translocation of **a** Probe-375 (10 kbp) and Probe-141 (38.5 kbp) on their own, **b** Probe-375 and Probe-141 in the presence of miR-375-p, **c** Probe-375 and Probe-141 in the presence of miR-141-3p, and **d** Probe-375 and Probe-141 in the presence of both miR-375-3p and miR-141-3p. Scale bar in (i): photon trace (green), vertical, 100 counts/ms, horizontal, 3 s; current trace, vertical, 30 pA, horizontal, 3 s. Scale bar in (ii): photon trace (green), vertical, 100 counts/ms, horizontal, 5 ms; current trace, vertical, 25 pA, horizontal, 5 ms. **e** Histograms summarising the ratio of synchronised events based on the data obtained from **a**–**d**. **f** Histograms for the synchronisation ratio for 3 miRNA populations. All DNA carriers and miRNAs were used at a concentration of 10 pM. The translocations were performed in 100 mM KCl buffer (5 mM MgCl_2_, 10 mM Tris-HCl, 1 mM EDTA, pH = 8.0) at an applied potential bias of −300 mV. Laser power was 90 ± 4 µW. The error bars in **e** and **f** represent the standard deviation measured from 3 different nanopore sensors (*n* = 3). Data are presented as mean ± s.d.

Minimal to no fluorescence was observed (MB was in the quenched state), in the photon-time traces for the molecular probes with no miRNA present in solution, Fig. 3a. In contrast, typical translocation events were observed in the electrical channel and were associated with the corresponding 10 and 38.5 kbp DNA probes. To quantify the binding of miRNAs, electrical events were firstly classified into two populations by analysing the dwell time and peak current area, Supplementary Fig. 7. The synchronisation ratio (*S*) is defined as the number of synchronised events relative to the total number of electrical events for a specific probe. With no miRNA present, *S* was very low: 0.8 ± 0.5% for Probe-375 and 0.9 ± 0.4% for Probe-141, Fig. 3e. Upon addition of an equimolar concentration of synthetic miR-375-3p, *S* increased to 76.1 ± 7.6% for Probe-375 whilst as expected the synchronisation ratio for Probe-141 remained low (*S* = 1.1 ± 0.3%), Fig. 3b, e. This indicates that only miR-375-3p successfully hybridised to Probe-375 and opened the MB ‘stem-loop’, enabling fluorescence emission. Experiments were repeated in the presence of miR-141-3p only, and the opposite trend was observed. Probe-141 had a high synchronisation ratio (*S* = 80.5 ± 9.2%), while Probe-375 were detectable only electrically with negligible *S*, Fig. 3c, e. The accuracy in discriminating these two miRNA populations was calculated to be 98.8% at picomolar concentrations. Final experiments were performed in the presence of both miR-375-3p and miR-141-3p, confirming that both miRNA sequences could be simultaneously bound to their respective molecular probes and distinguished, Fig. 3d, e. Once validation was completed for multiplexed detection of two samples, the complexity was increased by using 3 miRNA sequences (miR-141-3p, miR-375-3p, let-7a). In this case, the let-7a-specific MB was incorporated into a 5.6 kbp dsDNA (Probe-let-7a), Supplementary Fig. 6. Much like in the previous case in the absence of miRNAs, only electrical signals were observed with a very low synchronisation ratio (0.7 ± 0.5%, 2.0 ± 2.2% and 1.8 ± 1.9% for let-7a, miR-375-3p and miR-141-3p, respectively); however, in the presence of equimolar miRNAs, this percentage dramatically increased to 55.5 ± 9.5%, 75.9 ± 9.2% and 78.8 ± 7.6%, respectively, Fig. 3f and Supplementary Fig. 8. Further experiments were also performed to simultaneously detect miR-21 and let-7a as well as their corresponding DNA sequences, Supplementary Fig. 9.

### Quantification of the detection limits

**Fig. 4:**
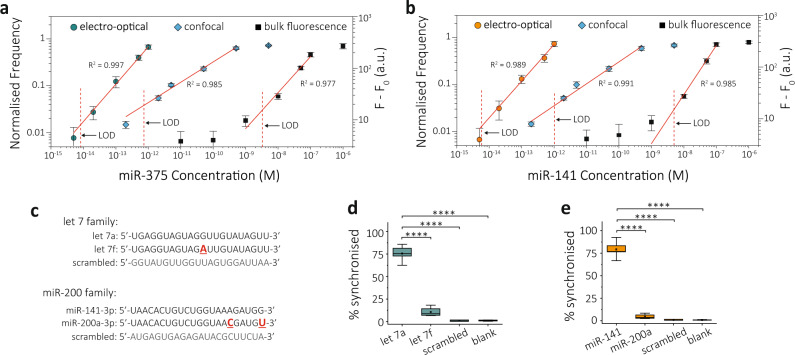
Sensitivity and selectivity of the detection platforms. **a**, **b** Calibration curves for miR-375-3p (**a**) and miR-141-3p (**b**) using asymmetric salt buffer (*cis*, 40 mM KCl; *trans*, 400 mM KCl). The concentrations of both molecular probes are 1 pM. As a comparison, data are shown using conventional single-molecule confocal microscopy and bulk fluorescence using molecular beacons at a concentration of 500 pM/100 nM. The dashed lines represent LODs at three times the standard deviation (3σ) of blank controls. The error bars represent 1 × σ obtained from 3 different nanopore measurements (*n* = 3). Data are presented as mean ± s.d. a.u., arbitrary units. **c** Sequences used for assessing the selectivity, consisting of the perfectly matched targets (let-7a, miR-141-3p), single-/double-nucleotide mutants (let-7f, miR-200a-3p), and scrambled sequences to Probe-let-7a and Probe-141. Variant bases are highlighted in red. **d**, **e** Box plots summarising the % synchronisation with these sequences. The black central line represents the median, the bottom and top edges mark the 25th and 75th percentiles, and the whiskers represent 1.5× interquartile range (IQR). Statistical significance was tested using a two-tailed Student’s *t*-test; ^****^P < 0.0001. All the measurements were performed with at least 3 independent nanopores (*n* = 5 for let-7a/miR-141-3p; *n* = 4 for let-7f/miR-200a; *n* = 3 for scrambled sequences; *n* = 5 for blank controls).

To quantify the target concentration, we performed a titration to assess the lower and upper limits of detection. Firstly, a calibration curve of *S* versus increasing concentration of synthetic miRNAs was obtained. In all cases, the concentration of Probe-375 or Probe-141 was kept constant at 10 pM, while the miRNA concentration was varied from 0.1 to 100 pM, Supplementary Figs. 10 and 11. A linear increase in *S* was observed for both miR-375-3p (*R*^2^ ≥ 0.998) and miR-141-3p (*R*^2^ ≥ 0.996) between 0.1 and 10 pM followed by the signal plateauing due to saturation of binding between the molecular probes and miRNA. The limit of detection (LOD), defined as three standard deviations (3σ) above background noise, was determined to be 0.13 and 0.1 pM for miR-375-3p and miR-141-3p, respectively. The ability to detect lower concentrations was limited by the low capture frequency of synchronised events associated with the decreased target miRNA concentrations. As demonstrated previously, a straightforward way to improve the capture rate is via the introduction of an asymmetric salt concentration across the nanopore^41^. To test this, we used a salt gradient of 40 mM/400 mM (*cis/trans*) and a lower molecular probe concentration of 1 pM. As shown in Supplementary Fig. 12, an almost 8-fold increase in capture rate was observed (from 0.15 ± 0.03 s^−1^ to 1.19 ± 0.12 s^−1^). This resulted in a significant improvement in the LOD, to 8 and 5 fM for miR-375-3p and miR-141-3p, respectively, Fig. 4a, b. Methods such as nanopore-based dielectrophoresis can likely be used to improve the detection limits even further^42^. As a comparison, we benchmarked our data against conventional confocal microscopy using identical detectors and bulk fluorescence measurements and obtained LODs of 0.67 pM/3.2 nM and 1.01 pM/5.1 nM for miR-375-3p and miR-141-3p, respectively, Fig. 4a, b and Supplementary Fig. 13.

### Single nucleotide sequence-specificity

Families of miRNAs share very high sequence homologies, often differing by a single nucleotide^43^. This, in combination with their short length, presents challenges for discrimination between family members, mainly when dealing with the high background interference present in clinical samples. To demonstrate the sequence-specificity of our sensor, we used miR-141-3p and let-7a and counterparts from within their families with high homologies: miR-200a-3p (90.9% homology to miR-141-3p) and let-7f (95.5% homology to let-7a), representing two and one nucleotide differences, respectively, Fig. 4c. miR-375-3p was not used as it has no close homologues (BlastN, NCBI). We assessed the specificity of both Probe-let-7a and Probe-141 (both at 10 pM) by incubating with equimolar amounts of either: (i) perfectly matching targets, let-7a and miR-141-3p, (ii) mismatched controls, let-7f and miR-200a-3p or (iii) scrambled sequences, Supplementary Fig. 14 (i–iii).

A high synchronisation ratio for molecular probes interacting with their corresponding perfectly matching targets, let-7a (75.6 ± 8.9%) and miR-141-3p (79.2 ± 9.5%) was observed, Fig. 4d, e. Single mismatch (let-7f) and double mismatch (miR-200a-3p) miRNAs showed significantly lower levels of synchronicity (*S* = 10.6 ± 5.3% (*P* < 0.0001) and *S* = 4.6 ± 2.6% (*P* < 0.0001)) while scrambled sequences exhibited equivalent fractions of synchronisation to carrier-only controls (0.88 ± 0.79% and 0.93 ± 0.61%, *P* < 0.0001), Fig. 4d, e. The significantly lower synchronicity of single and double mismatch-containing miRNAs was likely due to the lowered stability of the DNA–RNA duplex, preventing durable linearisation of the carrier ‘stem-loop’ hence maintaining the ‘closed’ form and quenching of the fluorophore. These results suggest our technique can differentiate miRNAs of very high sequence homology with excellent specificity, presenting possibilities also for the detection of single nucleotide polymorphisms.

### Direct miRNA sensing in serum

Sensing of biomarkers directly from unprocessed biological matrices such as blood is desirable to minimise the variations or bias originating from non-standardised multi-step pre-enrichment, extraction and purification steps^44^. Single-molecule analysis, directly from blood serum, also offers the unique advantage of detecting molecules regardless of their natural abundance, even without the need for any amplification step.

This was evidenced when conventional confocal microscopy was used to test the DNA carrier binding. Probe-375 and Probe-141, with equimolar concentrations of their corresponding target miRNAs, were spiked into a serial dilution of human serum at constant final carrier concentrations (10 pM each). We observed dramatic increases in background fluorescence in concordance with increasing human serum concentrations (1:20 to 1:2 serum:buffer, v:v) compared to that in 0.1 M KCl buffer, Supplementary Fig. 15a. The high background autofluorescence of the serum at any dilution completely obscured any single-molecule signatures from miRNA-bound carriers. However, by using a nanopipette, we observed a large decrease in background fluorescence, up to 50-fold for 1:2 serum:buffer ratio, Supplementary Fig. 15b. This marked improvement is largely due to the serum solution being confined to the nanopipette. The tip of the nanopipette is significantly smaller than the diffraction-limited probe volume, resulting in minimal background.

To test the stability and feasibility of electrical recordings in the bio-samples, we also recorded the ionic current as a function of serum concentration as well as in pure KCl buffer (0.1 M). As shown in Supplementary Fig. 15c, the baselines remained stable in pure KCl as well as in diluted serum (1:5 and below). Under these conditions, the nanopipettes were stable for >1 h (with >3 nanopipette tests). In principle, a higher dilution factor is preferable to maximise the stability of the electrical recordings; however, this limits the sensitivity. Hence, we selected a 1:10 ratio as the optimal dilution, as a compromise between sensitivity and quality of the signal.

### Multiplexed detection of miR-141-3p and miR-375-3p in prostate cancer patients

The utility and performance of our technology for PCa monitoring was examined by simultaneously quantifying multiple circulating miRNA expression levels directly from clinical samples (i.e. serum). As shown in Fig. 1a, the workflow is simple: serum collected from patients was mixed with the prepared DNA carrier-encoded molecular probes at the optimal serum:buffer ratio. Approximately 1 μl of this serum–carrier mixture was loaded into the nanopipette, and miRNA detection was subsequently multiplexed using our electro-optical sensing platform, see ‘Methods’ for details.

**Fig. 5:**
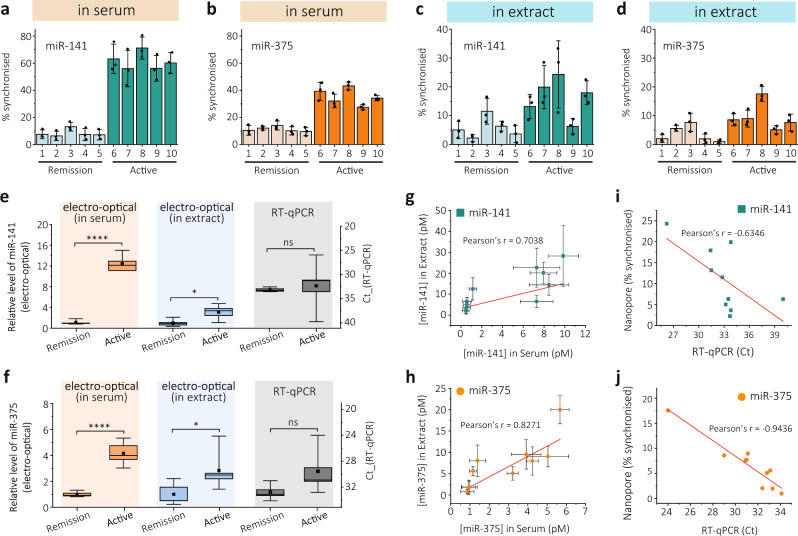
miRNA profiling of prostate cancer patients. **a**–**d** Synchronisation ratio of miR-141-3p and miR-375-3p for patients in remission (*n* = 5) and patients with active prostate cancer (*n* = 5) detected directly from serum (**a**, **b**) and in RNA extracted from serum (**c**, **d**). Error bars represent 1 × σ obtained from 3 different nanopore measurements (*n* = 3). **e**, **f** Box plots of relative expression levels for miR-141-3p (**e**) and miR-375-3p (**f**) for patients in remission and with active cancer detected directly from serum, RNA extract and RT-qPCR (remission: *n* = 5; active: *n* = 5), respectively. For all boxes, the black central line represents the median, the square represents the mean, the bottom and top edges mark the 25th and 75th percentiles. The whiskers denote the intervals between 5th and 95th percentiles. Statistical significance was tested using two-tailed Student’s *t*-test. For miR-141-3p, ^****^P < 0.0001; ^*^P = 0.0166; ns, not significant, *P* = 0.724. For miR-375-3p, ^****^P < 0.0001; ^*^P = 0.0488; ns, not significant, *P* = 0.079. **g**, **h** Correlation between expression values measured in serum and in extracts for miR-141-3p (**g**) and miR-375-3p (**h**). Pearson’s *r* for miR-141-3p, *r* = 0.7038, *P* = 0.0231, and for miR-375-3p, *r* = 0.8217, *P* = 0.0035. Concentrations of miRNAs were calculated from the calibration curves in Fig. 4 and Supplementary Fig. 11. The error bars represent 1 × σ obtained from 3 different nanopore measurements (*n* = 3). **i**, **j** Correlation between % synchronised measured in extracts and Ct value measured using RT-qPCR for miR-141-3p (**i**) and miR-375-3p (**j**), respectively. Pearson’s *r* for miR-141-3p, *r* = −0.6346, *P* = 0.0485, and for miR-375-3p, *r* = −0.9436, *P* = 0.0001. Statistical significance in **g**–**j** were tested using two-tailed Student’s *t*-test.

Expression levels of miR-141-3p and miR-375-3p were initially measured in blood serum from patients with active PCa (*n* = 5) and patients in remission (*n* = 5). We observed an elevated frequency of synchronised electro-optical events in the active cancer cohort, indicating higher levels of miR-141-3p and miR-375-3p in circulation, Fig. 5a, b and Supplementary Fig. 16. To quantify the relative expression level, we calculated the concentration of each miRNA using the calibration curves shown in Fig. 4, and normalised the average level of remission cohort to 1, Fig. 5e, f. We calculated that the average relative expression levels of miR-141-3p and miR-375-3p for the active PCa cohort (*n* = 5) were greater than those for people who are in remission (*n* = 5) by 12.5 ± 1.6 and 4.2 ± 0.9 fold, respectively, Fig. 5e, f. The *P*-value was less than 0.0001, confirming that we can differentiate the active cancer cohort from the remission cohort with sensitivity and specificity of 100% using either miR-141-3p or miR-375-3p, Fig. 5a, b. As a comparison and to benchmark the results, we also used the same technology to analyse miRNAs extracted from serum. Total RNA was extracted from 0.5 ml of serum samples using the Monarch Total RNA Miniprep Kit (see ‘Methods’). We observed similar trends of increased frequency of detected events for miR-141-3p and miR-375-3p in patients with active disease compared to those in remission, Fig. 5c, d and Supplementary Fig. 17. However, the difference between active and remission cohorts was comparatively smaller (3.1× and 2.8× for miR-141-3p and miR-375-3p, respectively) and less significant, statistically (miR-141-3p, *P* = 0.0166; miR-375-3p, *P* = 0.0488), than results obtained directly from serum, Fig. 5e, f. The sensitivity values obtained for miR-141-3p and miR-375-3p were also lower: 80% and 60%, respectively, at 100% specificity. In order to compare our technique against the current gold standard for miRNA analysis, we performed RT-qPCR for both miR-141-3p and miR-375-3p. Although patients with active disease trended towards higher levels of both miRNAs (2^-∆Ct^; miR-141-3p, 4.3×; miR-375-3p, 7.9×), it was less significant (miR-141-3p, *P* = 0.724; miR-375-3p, *P* = 0.079), Fig. 5e, f.

We also tested the correlation of miRNA levels detected between the extracts and the serum, Fig. 5g, h. Both miR-141-3p and miR-375-3p show moderate correlation between the two sample types, with Pearson’s *r* of 0.7038 (*P* = 0.0231) and 0.8217 (*P* = 0.0035), respectively. The discrepancy in the correlation could result in part from some level of RNA loss or degradation during the extraction process, which also contributes to the greater variability of miRNA expression level detected. The discrepancy may also be attributable to the fact that only freely circulating or some protein-bound miRNAs can be detected directly from the serum, while detection in the extracts could also include those encapsulated in exosomes/vesicles^45^. This was evidenced by the generally lower average concentrations in serum within individual patients, Supplementary Fig. 18. Further experiments are underway to examine this impact by lysing the exosomes/vesicles using chemicals to release miRNAs and detect directly without extractions.

The results from RT-qPCR and nanopore from extracted RNA also show moderate correlations for miR-141-3p and miR-375-3p (with Pearson’s *r* of −0.6346 and −0.9436, *P* = 0.0485 and 0.0001, respectively), Fig. 5i, j. During RT-qPCR, the error-prone enzyme-based reverse transcription and amplification steps also contribute to greater variability and lower accuracy. The results from RT-qPCR exhibit worse correlations with those directly from serum using nanopores, Supplementary Fig. 19. It should also be noted that, when using extracted RNA, a normalisation step such as spiking-in a standard non-human miRNA, e.g. cel-miR-39, is usually needed to evaluate the loss of RNA during purification^46^. However, it is not clear whether all the miRNAs extracted from biofluids share the same efficiency, or whether the spike-in miRNA in blood is as stable as the endogenous ones. Therefore, the ability to assay miRNAs in parallel, directly from biological samples, is even more desirable, as variabilities between individual tests would be largely minimised.

### Profiling a panel of 3 miRNAs for cancer stage classification

**Fig. 6:**
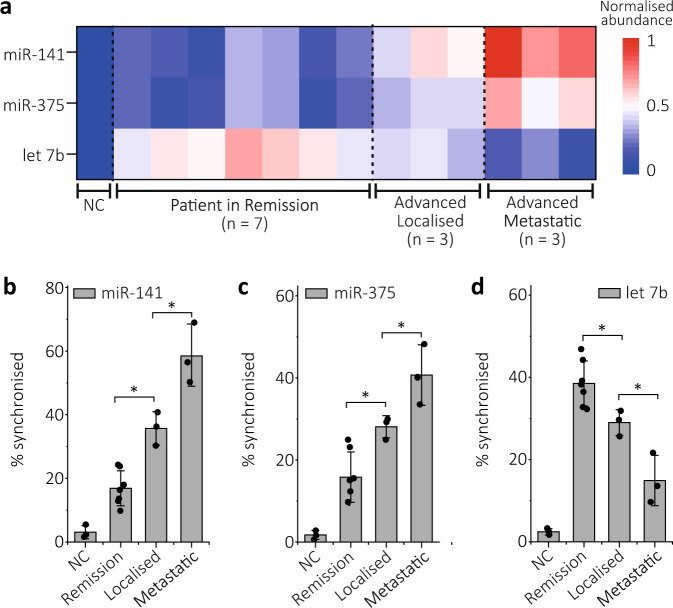
Mapping of 3 miRNAs to classify prostate cancer stages. **a** Heat map of two up-regulated (miR-141-3p & miR-375-3p) and one down-regulated (let-7b) miRNAs in prostate cancer patients with advanced localised (*n* = 3), metastatic (*n* = 3) disease and patients in remission (*n* = 7). NC, negative control. **b**–**d** Relative expression levels of **b** miR-141-3p, **c** miR-375-3p and **d** let-7b in negative controls, remission patients, advanced localised and metastatic disease patients. Values described were obtained from 3 technical replicates. Data are presented as mean ± s.d. Statistical significance was tested using two-tailed Student’s *t*-test; ^*^P < 0.05. For localised disease, *P* = 0.001, 0.0117 and 0.0246 for miR-141-3p, miR-375-3p and let-7b, respectively. For metastatic, *P* = 0.0222, 0.0497 and 0.0238, for miR-141-3p, miR-375-3p and let-7b, respectively.

The possibility to classify cancer stages by simultaneously detecting a panel of miRNAs directly from patients’ serum was also evaluated. To this end, we selected miR-141-3p and miR-375-3p, which are up-regulated for PCa, and let-7b, which is found to be down-regulated.^47^ Single-molecule electro-optical experiments were then performed with negative controls (no targets) and PCa patients’ serum sourced from cohorts of patients in remission (*n* = 7), with advanced localised (*n* = 3) and metastatic (*n* = 3) disease, Supplementary Fig. 20. We observed a progressive change in the expression patterns for the 3 miRNAs in individuals with different stages, Fig. 6a. The expression levels of miR-141-3p and miR-375-3p were observed to be up-regulated in localised PCa between patients in remission and those with advanced localised disease (*P* < 0.05), and a further increase was also observed in metastatic disease patients (*P* < 0.05), Fig. 6b, c. Meanwhile, the expression level of let-7b was observed to be down-regulated in advanced PCa, Fig. 6d. Interestingly, we can differentiate localised and metastatic PCa diseases with moderate significance (*P* < 0.05) although only limited patient sizes were tested.

## Discussion

In summary, we demonstrated that electro-optical nanopores and size-coded molecular probes could be efficiently used for a single-molecule, extraction- and amplification-free multiplexed miRNA detection from human serum. Our platform was able to distinguish PCa patients in remission from those with active disease with 98.8% selectivity via simultaneous expression profiling of two significant miRNAs directly from unextracted clinical samples. We also demonstrated the possibility to classify cancer stages by simultaneously profiling a panel of 3 miRNAs. This technique showed superior sensitivity with almost six orders of magnitude lower detection limit compared to bulk measurements and excellent sequence-specific selectivity, able to discriminate miRNAs from the same family with a single nucleotide difference.

Femtomolar sensitivity is essential for direct profiling of unamplified miRNA expression, particularly in biofluids where the concentrations of individual miRNAs are relatively low (typically ≤sub-nanomolar)^48^. Considering a fraction of miRNAs are encapsulated in exosomes or bound to protein complexes^45^, the absolute concentration of freely circulating biomarkers that can be directly detected in serum is even lower, and thus much more sensitive tools are required. Previous nanopore-based studies reported sensitivities primarily in the nM range, with a few at pM range, which are insufficient for direct detection in biofluids and therefore requires multiple pre-enrichment or concentration steps^29,31,49,50^. The fM sensitivity reported here represents a major improvement over current single-molecule^21,51^ or general electrochemical and fluorescence-based^35^ techniques and is comparable to amplification-based RT-qPCR^52^. Due to the single-molecule resolution, as little as 5 zeptomol (ca. 10^3^ copies) of miRNA could be accurately quantitated using this electro-optical multiplexed platform with a tiny sample volume (≈0.1 μl), which is more than 2000 times smaller than that typically required for RT-qPCR, assuming a minimum of 200 µl extracted serum, Supplementary Table 2.

The multiplexed capability could be readily expanded by encoding longer lengths of DNA as carriers. The primary limiting factor is the electrical resolution of DNA with nanopore. This study has shown that a <5 kbp shift in DNA carriers can be resolved with high confidence (Fig. 2c), indicating that multiplexing of ≈10 targets could be feasible with the length of λ-DNA (48.5 kbp). Furthermore, the resolution can be improved via the reconstruction of nanopores^53,54^, using higher bandwidth^55^ or smaller pore size^56^. In addition, the capability can be multiplied by encoding multi-colour fluorophores to the carriers.

We also tested the binding dynamics of miRNAs with the DNA carriers and found that 25–30 min is sufficient to reach full saturation of both miRNAs, Supplementary Fig. 21 and Supplementary Note 1. Assuming a minimum of 250 events needed for a >90% confident assay (Ref. ^29^) and the capture rates as mentioned above (Supplementary Fig. 12), we estimated that a maximum of 10 min is needed for the detection. This means that the turnaround time of our platform for multiple miRNA targets could be as short as 80 min (<1.5 h), while several hours to days are often needed for RT-qPCR^57^, microarray^58^ and RNA-Seq^59^, Supplementary Table 2. This is a significant advantage over existing techniques in the detection of critical miRNA populations with varying and short half-life^60^.

Based on these findings, we anticipate that the electro-optical technique could be readily used in real-world diagnostic applications, for example, biomarker validation or in the screening and monitoring of early-stage diseases by quantifying a class of significant miRNA biomarkers. With further optimisations and more clinical trials in whole blood, the small sample volume required and quick turnaround time may enable the use of blood collected from a finger-prick, decreasing associated costs and logistical complications associated with a traditional hospital or GP-based blood collection. Moreover, as the MB sequences can be designed as needed, and aptamers can be incorporated as demonstrated previously^33^, the targets are not restricted to miRNAs. One can perform the assay with multiple types of biomarkers simultaneously, for example, with other RNA, cfDNA or proteins, to further improve the accuracy and diagnostic value. As multiple targets can be identified simultaneously, the technology can potentially enable multiplexed detection of specific targets across several diseases, enabling possible usage in a generalised single screening test for multiple morbidities. This may lead to reduced associated costs and future lowering of the barrier to implementation of screening programmes^61^.

## Methods

### Preparation of 10 and 38.5 kbp DNA carriers

Commercial λ-DNA (48.5 kbp, 500 μg/ml) (New England Biolabs) was digested into two segments, 10 and 38.5 kbp, using the restriction enzyme ApaI (50,000 U/ml, New England Biolabs), according to the supplier’s protocol. Briefly, 12.5 μl of stock λ-DNA (15.8 nM), 5 μl of 10× CutSmart buffer, 2.5 μl ApaI and 30 μl of nuclease-free water (Sigma-Aldrich) were mixed to a final volume of 50 μl. This mixture was incubated at 25 °C for 30 min to allow digestion, then 65 °C for 20 min for heat inactivation of enzymes.

For the preparation of Probe-375 (10 kbp) and Probe-141 (38.5 kbp), 25 μl of 400 nM MB_miR-375 (to the 10 kbp fragment, 5ʹ-AGGTCGCCGCCC T(Alexa 488) CCGTACG T CAC GCG AGC CGA ACG AAC AAA CGTACGGA-Dabcyl-3ʹ) and 25 μl of 400 nM MB_miR-141 (to the 38.5 kbp fragment, 5ʹ-GGGCGGCGACCT T(Alexa 488) CCGGCAC C CAT CTT TAC CAG ACA GTG TTA GTGCCGGA-Dabcyl-3ʹ) were added to the Apa I digested λ–DNA (50 μl) in a fragment-to-MB ratio of 1:50, Supplementary Fig. 5. The hybridisation was run using a PCR annealing device (TC-3000, TECHNE) with a customised protocol. In brief, the mixture was heated to 75 °C for 5 min to denature the MB oligos and DNA fragments that were digested from λ-DNA followed by the annealing procedure by decreasing the temperature to 15 °C at a rate of 1 °C per min. The resulting products were held at 4 °C before purification.

Unbound MB oligos, ApaI and BSA from the CutSmart buffer were removed from the solution using a 100 kDa MWCO Amicon Ultra Filter (Millipore) according to the manufacturer’s instructions. Briefly, the solution was transferred into the filter column, and a buffer solution (100 mM KCl, 5 mM MgCl_2_ in 1× TE (pH = 8.0)) was added to a total volume of 400 μl. The purification was then operated with six cycles of ultra-centrifuging at a speed of 3500*g* at 4 °C for 30 min. The resulting product was recovered by inversion of the filter column and subsequent centrifugation at 1000*g* for 2 min. The final concentrations of carriers (both 10 and 38.5 kbp) were determined via Nanodrop 2000c (Thermo Fisher Scientific), measuring the UV-Vis absorbance at 260 nm (see Supplementary Fig. 5d) and stored at −20 °C before use.

The preparation of Carrier-let-7a (10 kbp) and Carrier-21 (38.5 kbp) follow the same procedure as described above only replacing the MB sequences with corresponding MB sequences specific for let-7a and miR-21 (sequences provided in Supplementary Table 1).

### Preparation of 5.6 kbp DNA carrier

Next, 5.6 kbp DNA was obtained from the BstE II digestion of λ-DNA (New England Biolabs). To prepare Carrier-let-7a (5.6 kbp), MB sequence that target let-7a (5ʹ-AGGTCGCCGCCC T(Alexa 488) CCGTACG T CAC GCG AGC CGA ACG AAC AAA CGTACGGA-Dabcyl-3ʹ) was firstly incubated with the BstE II digested product at the molar ratio of 100:1 and annealed using the same protocol as described above. The MB-engineered DNA carrier was then isolated using a 0.7% (wt%) agarose gel electrophoresis (Supplementary Fig. 6) and extracted using Monarch DNA Gel Extraction Kit (New England Biolabs) according to the supplier’s protocol. The concentration of the DNA carrier probe was determined using Nanodrop, and the resultant solution was stored at −20 °C before use.

### Serum preparation

Whole blood from patients who attended clinics at Imperial College Healthcare NHS Trust (London, UK) with different stages of PCa was collected in 2014 following written patient consent using a standard venipuncture procedure and stored as a subcollection in the Imperial College Healthcare Tissue Bank of Imperial College Healthcare NHS Trust (London, UK) and Imperial College London. The study that related to human participants has been approved by Wales Research Ethics Committees (RECs). To prepare the serum, the blood in a red-topped Vacutainer (silicon-coated with clot activator BD) was kept upright at room temperature for 30–60 min, allowing the blood to clot. Samples were then centrifuged at 1000–3000*g* at room temperature for 10 min, and the serum in the supernatant was isolated, aliquoted (1 ml per cryovial) and stored at −80 °C. Prior to use, the serum was thawed entirely at room temperature for approximately 1 h.

### Serum RNA extraction

Total RNA was extracted from 0.5 ml of serum samples using the Monarch Total RNA Miniprep Kit (T2010S, NEB) according to the manufacturer’s instructions. Samples were eluted in 50 μl nuclease-free water and total RNA concentration determined via Nanodrop D-1000 (Thermo Scientific). Extracted samples were aliquoted and stored at −20 °C.

### RT-PCR assay

cDNA synthesis and qPCR were performed using a miRCURY LNA Universal RT microRNA PCR system (Exiqon) according to the manufacturer’s protocol. Two microlitre of RNA extract was reverse transcribed in a 10 μl final reaction volume using Universal cDNA Synthesis Kit ll (Exiqon). The resulting cDNA was diluted in nuclease-free water containing ROX passive reference dye (Invitrogen) at a final concentration of 500 nM. Individual qPCR was performed on diluted cDNA using the ExiLENT SYBR Greenmastermix (Exiqon) and LNAPCR primers (Exiqon) for miR-141-3p and miR-375-3p. Real-time PCR amplification was performed in duplicate on an Applied Biosystems 7900HT system using a cycle condition according to the template file (available at www.exiqon.com/sds).

### Bulk fluorescence measurement

All MBs were firstly hybridised with a 10× of short cDNA oligos (BTA1 or BTA2, sequences can be found in Supplementary Table 1) that have 12-base complementary to the tail of MB to mimic the attachment to the 12-base sticky overhang of DNA carrier. The MB–cDNA complex was then diluted using 0.1 M KCl buffer (5 mM MgCl_2_, 10 Tris-HCl, 1 mM EDTA, pH = 8.0) and incubated with various concentrations of corresponding miRNA targets at a final MB concentration of 100 nM. The incubation time was normally about 45 min unless stated otherwise. The fluorescence emission was recorded from 480 to 600 nm using Cary Eclipse Fluorescence Spectrophotometer (Agilent Technologies) with 460 nm excitation. All emission spectra were subtracted by the buffer-only control to obtain the fluorescence emission spectrum. Quantitative fluorescence emission values used throughout are fluorescence intensities recorded at λ_em_ = 515 nm.

### Preparation of nanopipettes

All the nanopipettes were fabricated by pulling quartz capillaries (GQF100-50-7.5, World Precision Instruments, UK) using a laser-assisted pipette puller (Sutter Instrument, P-2000, USA) as per protocol reported previously by our group^33,62^. Prior to pulling, the capillaries (inner diameter: 0.5 mm, outer diameter: 1.0 mm, length: 7.5 cm) were cleaned thoroughly for approximately 30 min using a plasma cleaner (Harrick Plasma) to remove any organic residues or contaminants on the quartz surface. A capillary was then set up on the capillary holder of the puller and pulled using an optimised two-line protocol: (1) HEAT: 775; FIL: 4; VEL: 30; DEL: 170; PUL: 80, (2) HEAT: 825; FIL: 3; VEL: 20; DEL: 145; PUL: 180. Nanopipettes generated using this protocol have pore size averaged at 22 ± 3 nm (*n* = 5), according to the SEM characterisation and conductance calculation, Supplementary Figs. 1 and 2. It is important to be aware that the pulling protocols are specific to the instrument and are very sensitive to environmental conditions such as humidity and temperature.

### Set-up of electro-optical nanopore sensing platform

All the optical measurements were carried out by a previously reported custom-built single-molecule confocal microscope^63,64^. Briefly, a 488 nm continuous laser beam (Sapphire 488LP, Coherent) was generated and expanded by a beam expander (ThorLabs). The laser beam was then introduced into an Olympus IX71 inverted microscope via a dichroic mirror. A 60× water immersion objective (1.20 NA, UPLSAPO 60XW, UIS2, Olympus) was used to introduce the laser beam to illuminate the orifice of nanopipette tip and collect the generated fluorescence. A 75 µm pinhole (P75S, ThorLabs) was used at the image focal plane to reject the light that is out of focus. The fluorescence was then split into two channels (500–580 nm and 640–680 nm) using a dichroic mirror (630 DCXR, ThorLabs) and detected using two avalanche photodiodes (APDs) (SPCM-AQR-14, PerkinElmer), respectively. Schematic illustration and details are shown in Supplementary Fig. 3a. The dimension of the confocal volume was estimated to be 1.94 ± 0.03 fl (*n* = 3) via measuring fluorescence correlation spectroscopy (FCS) using 70 pM Atto 488 (ThermoFisher) aqueous solution.

To achieve ideal synchronised electro-optical measurements, precise alignment between the nanopore orifice and the optical detection volume is required. Before alignment, a quartz nanopipette was mounted on a coverslip (Thickness Class No. 1.5, 24 × 50 mm) via a customised 3D-printed stage and was then set inside a customised Faraday cage with a sample holder on the base, Supplementary Fig. 3a–c. The alignment was achieved using a high-resolution motorised stage (Prior Scientific) with a minimum 10 nm step size and monitored using a sensitive emCCD camera (iXon Ultra, Andor). First, the *x*–*y* alignment was performed by carefully moving the sample holder to position the nanopipette tip orifice at the centre of the laser spot. This could be visually monitored using the live video of emCCD with the top lamp on (see Supplementary Fig. 3d). Then, the *z*-dimension was aligned by slowly lowering the *z* scan at a minimum step size (10–50 nm per step) with the top light turned off. This procedure was monitored by recording the photon trace in the red channel (640–800 nm) and was stopped when a sharp increase in the photon count was observed, as shown in Supplementary Fig. 4. The increase in signal was due to light scattering of the coverslip and nanopipette tip, as shown in Supplementary Fig. 3b. It should be mentioned that we observed minimum drift of nanopipette up to 2 h when fixed with this stage.

### Translocation experiments

Nanopipette translocation experiments were performed in an inside-to-outside (*cis*-to-*trans*) manner unless otherwise specified. In this work, ~1 μl of samples in electrolyte solution was backfilled into the nanopipette (*cis* chamber) using a MicroFil syringe and an Ag/AgCl electrode was inserted to serve as the working electrode. The nanopipette was then set up on a coverslip using a customised stage and fixed onto the confocal microscope with a customised Faraday cage. Another Ag/AgCl electrode was placed outside the nanopipette tip (*trans* chamber) acting as the reference electrode. Around 50 μl of electrolyte (100 or 400 mM KCl solution) was added to the coverslip surrounding the nanopipette tip to form an electrochemical cell. After appropriate alignment, translocation experiments were performed by applying potentials via an A&M 2400 patch-clamp amplifier (A-M Systems, Inc.) and the corresponding current was recorded using a WinWCP software. The synchronised optical signals were recorded using a custom-written Labview software.

### Translocation of MB-carriers with synthetic miRNAs/DNAs

Prior to translocation, the prepared MB-carriers were diluted using KCl buffer (100 mM KCl, 5 mM MgCl_2_, 10 mM Tris-HCl, 1 mM EDTA, pH = 8.0) and mixed with different concentration ratios of synthetic miRNAs/DNAs at a final carrier concentration of 10 pM to incubate at room temperature for 2 h unless otherwise stated.

### Translocation experiments in salt gradient

For the translocation experiments in salt gradient, the MB-carriers was diluted using 40 mM KCl (2 mM MgCl_2_, 4 mM Tris-HCl, 0.4 mM EDTA, pH = 8.0) and incubated with synthetic miRNAs at different concentration ratios to final carrier concentration of 1 pM. The buffer added to the outside nanopipette (*trans*) was 400 mM KCl (5 mM MgCl_2_, 10 mM Tris-HCl, 1 mM EDTA, pH = 8.0).

### Translocation of MB-carriers with clinical samples

Experiments with patients’ serum were performed under the salt gradient condition. MB-carriers were diluted using 40 mM KCl buffer (2 mM MgCl_2_, 4 mM Tris-HCl, 0.4 mM EDTA, pH = 8.0) and subsequently mixed with human serum at a reported ratio at final carrier concentration of 1 pM. The mixture was incubated at room temperature for 2 h prior to being introduced into the nanopore. About 50 μl of 400 mM KCl (5 mM MgCl_2_, 10 mM Tris-HCl, 1 mM EDTA, pH = 8.0) was added outside nanopipette and synchronised electro-optical measurements were then performed at a potential bias of −300 mV. For experiments with serum extracts, MB-carriers were diluted using 100 mM KCl buffer (5 mM MgCl_2_, 10 mM Tris-HCl, 1 mM EDTA, pH = 8.0) and subsequently mixed with the extracts at a ratio of 100:1 at final carrier concentration of 10 pM. After incubation for 2 h, the mixture was introduced into the nanopore, and synchronised electro-optical measurements were performed.

### Data acquisition and statistical analysis

A NI 6602 DAQ card (National Instruments) was coupled with the APDs for obtaining the optical data while another NI-USB 6251 DAQ card (National Instruments) was used for the electrical data collection. The synchronisation of electrical and optical detection was achieved through a connection between these two cards and triggered via the application of a TTL pulse using a custom-written Labview program (2014 SP1, 64-bit). All the electrical signals were sampled at 100 kHz and filtered at 10 kHz using a low-pass Bessel filter. Optical photon counts were collected via APD detectors with a time resolution of 10 μs. A custom-written application in Matlab (R2019b) was used to analyse synchronised optical-electrical events. Electrical and optical data were initially analysed independently using this application. The obtained optical and electrical events were then further analysed using a Coincident App based on Matlab. Briefly, (1) the raw electrical data were loaded and filtered at 10 kHz while the optical data were resampled at 1 ms. (2) Track and subtract the baseline of the electrical trace. This step is not necessary for optical data and hence is omitted. (3) A histogram of the baseline-subtracted ionic current trace (or photon trace) is obtained, and the open pore current (or background photon rate) is then fitted with a Poisson probability distribution function. A cut-off threshold of 7× (5× for optical data) standard deviations above the mean noise (background) level are applied to define the event boundaries. (4) Parameters such as peak maximum, total peak width and peak current area are extracted. The translocation times were determined by using the FWHM (typically 3) of the DNA carrier signal. (5) A custom script was used to define the synchronised electro-optical events by cross-correlating their time stamps and obtain the statistics of the synchronisation ratio. All mean values reported were obtained from the average of the data set, and all errors represent one standard deviation. Plots and charts were performed using Origin 2019.

### Reporting summary

Further information on research design is available in the Nature Research Reporting Summary linked to this article.

## Supplementary information

Supplementary Information

Reporting Summary

## Data Availability

The data that support the plots within this paper and other findings of this study are available from the corresponding author upon reasonable request. Source data for the figures and supplementary figures are provided as a Source Data file.

## Code availability

The Nanopore App used to analyse the data is available from the corresponding author upon reasonable request.
