# Supplementary material for: Single-molecule amplification-free multiplexed detection of circulating microRNA cancer biomarkers from serum
Source: Nat Commun. 2021 Jun 10;12:3515. doi: 10.1038/s41467-021-23497-y (PMC8192752; doi:10.1038/s41467-021-23497-y)
Supplement: Supplementary file 1 — Supplementary Information [file 41467_2021_23497_MOESM1_ESM.pdf]

Supplementary Information

Single-molecule amplification-free multiplexed detection of circulating microRNA cancer biomarkers from serum

Shenglin Cai[1], Thomas Pataillot-Meakin[1,2,3], Akifumi Shibakawa[3], Charlotte L. Bevan[3,]*, Sylvain Ladame[2,]*, Aleksandar P. Ivanov[1,]*, Joshua B. Edel[1,]*

[1]Department of Chemistry, Imperial College London, Molecular Science Research Hub, White City Campus, 82 Wood Lane, London W12 0BZ, U.K.
[2]Department of Bioengineering, Imperial College London, Sir Michael Uren Hub, White City Campus, 86 Wood Lane, London W12 0BZ, U.K
[3]Department of Surgery and Cancer, Imperial College London, Hammersmith Hospital, London, United Kingdom

Correspondence to: charlotte.bevan@imperial.ac.uk; s.ladame@imperial.ac.uk; alex.ivanov@imperial.ac.uk; joshua.edel@imperial.ac.uk

Supplementary Methods

UV-Vis measurements

The concentration and purity of newly ordered DNA/RNA oligos and in-house prepared MB-engineered DNA Carriers were estimated by measuring the UV-Vis absorbance using a Nanodrop (Thermo Scientific). 1 µl of DNA/RNA solution was loaded onto the pedestal of the Nanodrop and absorbance was measured from 220 to 350 nm. The concentrations of oligos was determined using the absorbance value at 260 nm. To estimate the DNA carrier concentrations, we assumed fragment 1 (10 kbp) and fragment 2 (38.5 kbp) to be equal in molarity. A typical UV-Vis absorbance spectrum of the DNA MB-carrier is shown in Supplementary Fig. 4d. The purity of the obtained carrier was assessed by calculating the ratio of $A_{260/280}$ and $A_{266/230}$. Typically, the value of $A_{260/280}$ and $A_{266/230}$ calculated are 1.90 ± 0.3 and 2.03 ± 0.36 (Supplementary Fig. 4d), which indicate high purity of DNA carrier samples.

Gel electrophoresis for characterisation

DNA digestion was characterised by gel electrophoresis. Briefly, 10 µl of different concentrations of DNA digestion (ranging from 0.125 to 0.375 ng/µl) was firstly stained using 2 µl of 6 × purple loading dye (New England BioLabs, UK). 10 µl of the stained DNA and 1 kbp extended DNA ladder were loaded into the well of a 6.0% agarose gel. The electrophoresis was performed in TBE buffer at a potential of 4 V/cm for 180 min. Then, the gel was stained with 25 ml of 1 × SYBR Gold solution and incubated for 30 min under darkness. The gel was visualised using a Gel-Bright ™ LED Light Box (Biotium ™) and pictured using a camera.

Supplementary Figures

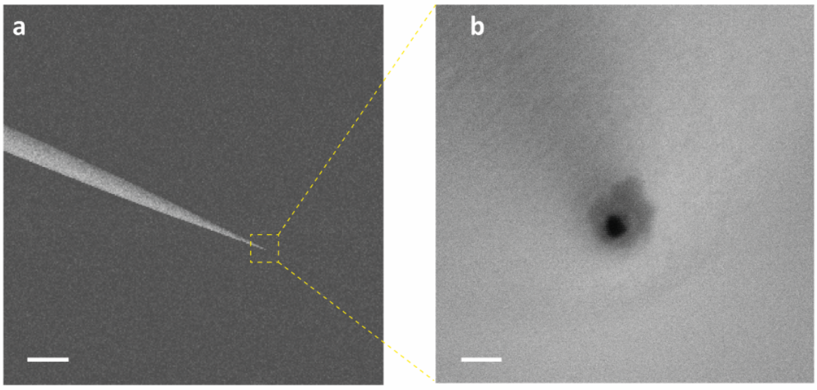

Supplementary Fig. 1 | SEM images of a representative nanopipette.

(a) Side view SEM image showing the tip of nanopipette pulled from a quartz capillary. The pipette has a conical shape and tapered tip. (scale bar: 20 µm) **(b)** A close-up SEM image of the cross-section of nanopipette (scale bar: 50 nm). The diameter of nanopipettes measured by SEM is 22 ± 3 nm (n = 5).

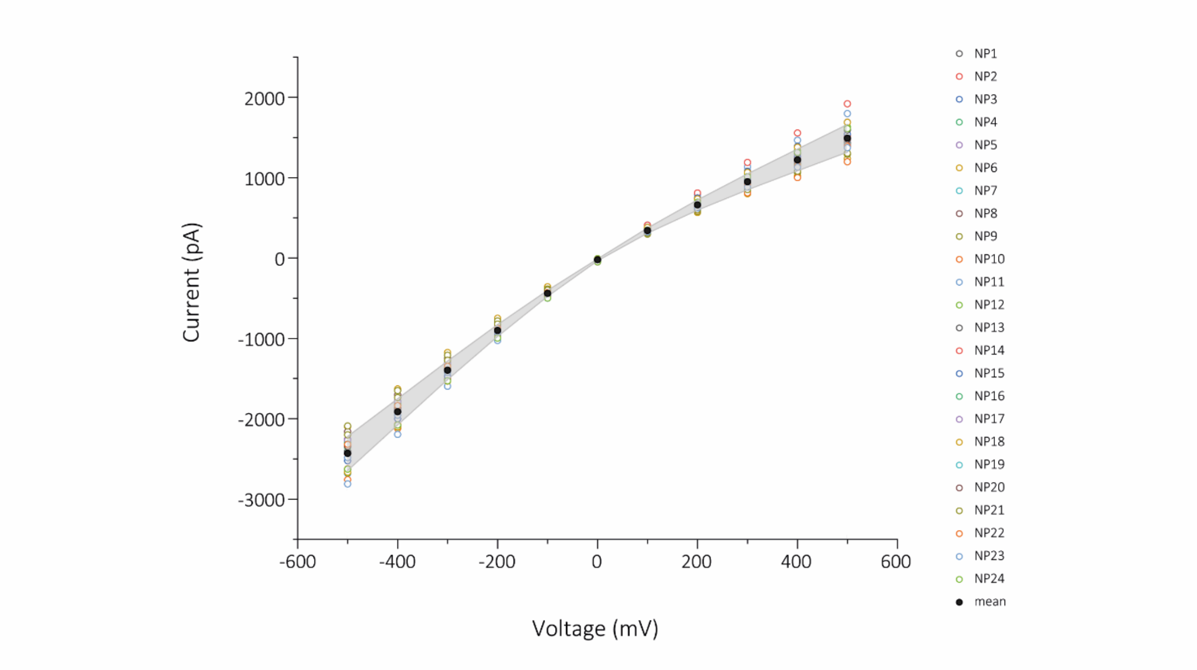

Supplementary Fig. 2 | Current-voltage (I-V) curves of nanopores.

The I-V characteristics of nanopores were performed before each experiment in 100 mM KCl (5 mM $MgCl_2$, 10 mM Tris-HCl, 1 mM EDTA, pH = 8.0). From the I-V curves, the nanopore conductance was calculated from the fit of the ohmic region (-100 to 100 mV), as G = 3.9 ± 0.2 nS (n = 24). The grey shaded region indicates the standard deviation (n = 24). The centre of error band represent the mean value of 24 measurements.

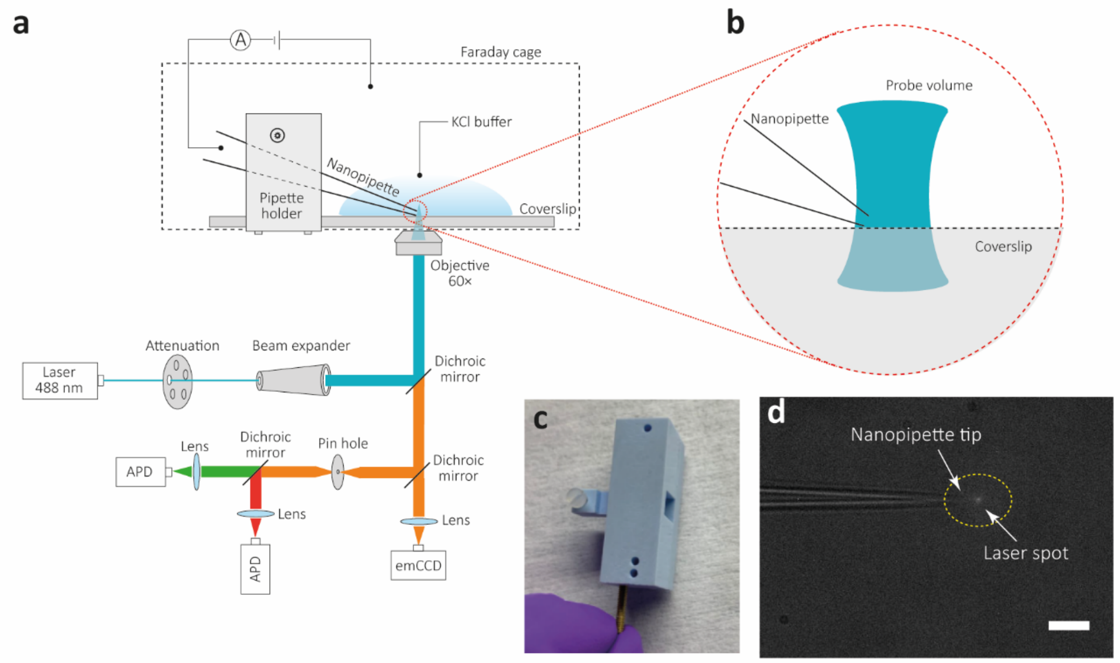

Supplementary Fig. 3 | Schematics of electro-optical nanopore sensing platform.

(a) Schematic of experimental setup using a custom-built single-molecule confocal microscope.[1, 2] The nanopipette was fixed on a 3D-printed pipette holder and was then set up inside a Faraday cage mounted on the microscope stage. **(b)** A schematic of the nanopipette tip alignment to the confocal probe volume. **(c)** A picture of the 3D printed nanopipette holder. **(d)** Bright-field image of the nanopipette aligned with the laser beam captured with an iXon Ultra emCCD camera (Andor, Oxford Instruments) (scale bar: 10 µm). Experiments were repeated for at least 3 times (n ≥ 3).

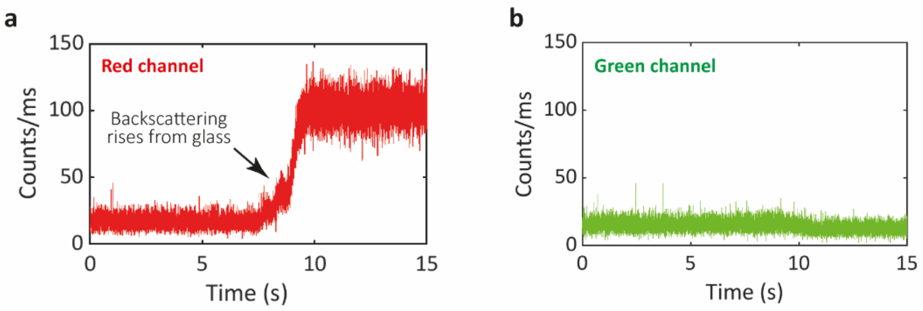

Supplementary Fig. 4 | Photo traces during alignment of the z-axis.

Photon–time traces recorded by avalanche photodiodes (APDs) from the **(a)** red (500-580 nm) and **(b)** green (640-680 nm) channel during the alignment of the z-axis. A sharp increase in backscattering was observed in the red channel as the confocal volume overlapped with the coverslip, and this reading was used as Z=0 reference point. At this position, a minimal change of photon counts was observed in the green channel. All the measurements were performed in 100 mM KCl buffer (5 mM $MgCl_2$, 10 mM Tris-HCl, 1 mM EDTA, pH = 8.0).

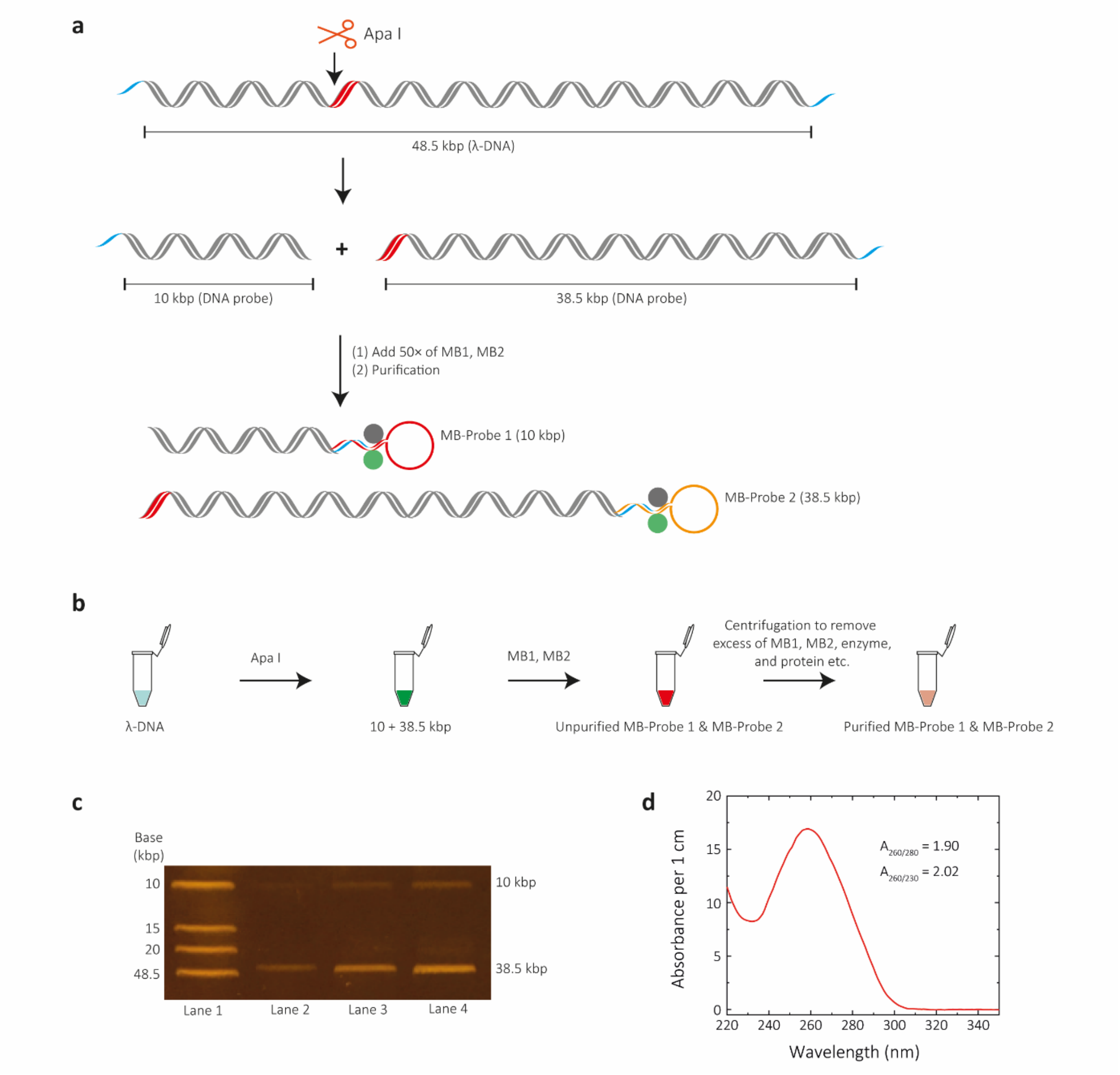

Supplementary Fig. 5 | Preparation and characterisation of MB engineered 10 and 38.5 kbp DNA molecular probes (MB-Probes).

(a) Schematic showing the Apa I digestion of λ-DNA into 10 and 38.5 kbp fragments and their subsequent assembly of MB probes. **(b)** Schematic illustrating the workflow of the preparation and purification of the 10 and 38.5 kbp DNA carrier encoded MB probes. The excess of MBs, as well as the protein in the buffer, were removed using 100 kDa MWCO Amicon ultrafilter. **(c)** Gel electrophoresis of the Apa I digested λ-DNA. Lane 1 shows the 1 kbp extend DNA ladder. Lane 2 to 4 were digestion of λ-DNA with a mass of 1.25, 2.5, and 3.75 ng, respectively. Experiments were repeated for at least 3 times (n ≥3). **(d)** UV-Vis spectrum for the DNA carrier probes in the presence of 100 mM KCl buffer (5 mM $MgCl_2$, 10 mM Tris-HCl, 1 mM EDTA, pH = 8.0).

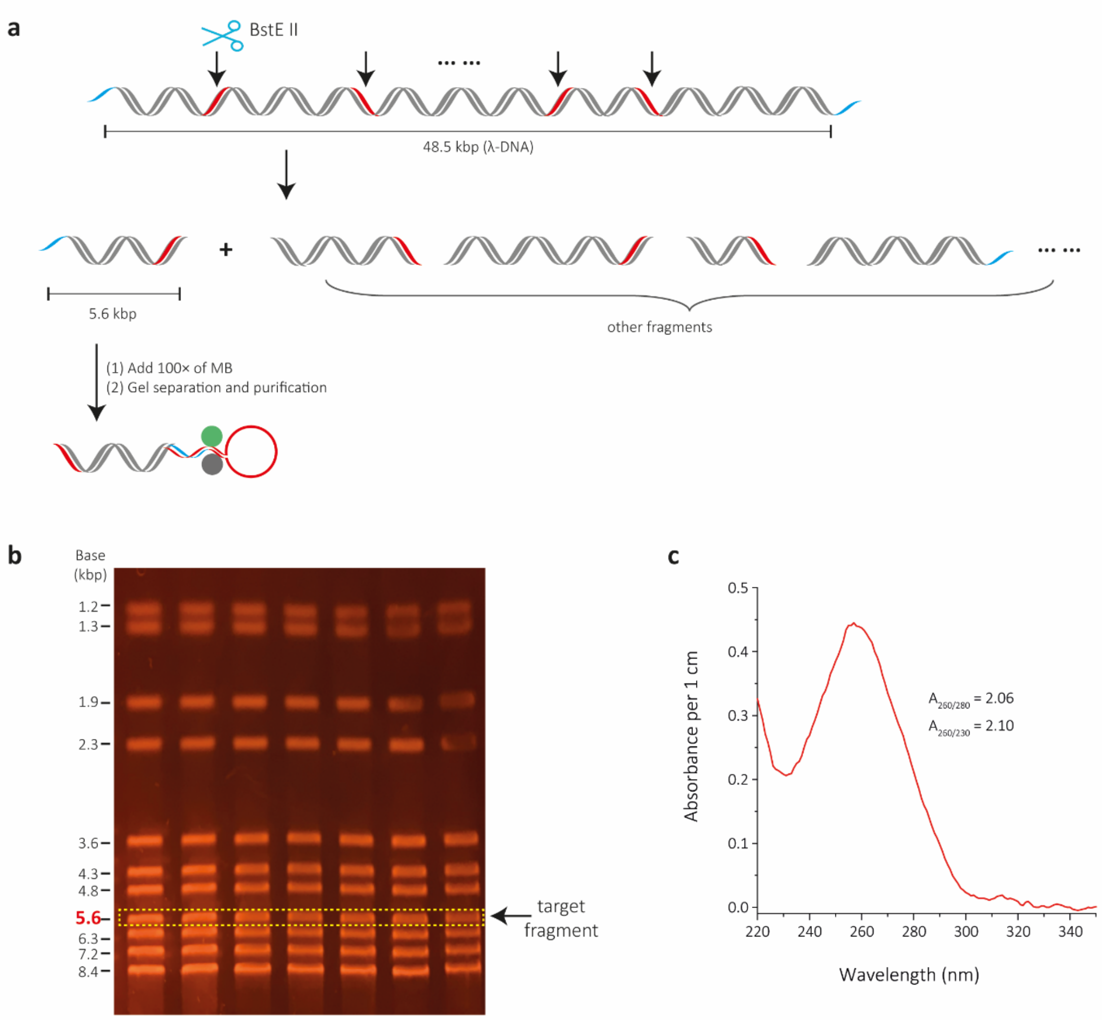

Supplementary Fig. 6 | Preparation of 5.6 kbp DNA encode MB probe from BstE II digested lambda-DNA.

(a) Schematic of BstE II digestion of λ-DNA. One terminal fragment with 5.6 kbp was hybridised with corresponding MB acting as the DNA carrier. **(b)** Gel electrophoresis separation of the 5.6 kb DNA probe. The target band was cut, and DNA was extracted using a DNA gel extraction kit (New England Biolabs). Experiments were repeated for at least 5 times (n ≥ 5). **(c)** Measurement of the concentration of the extracted DNA was performed using UV-Vis Spectroscopy

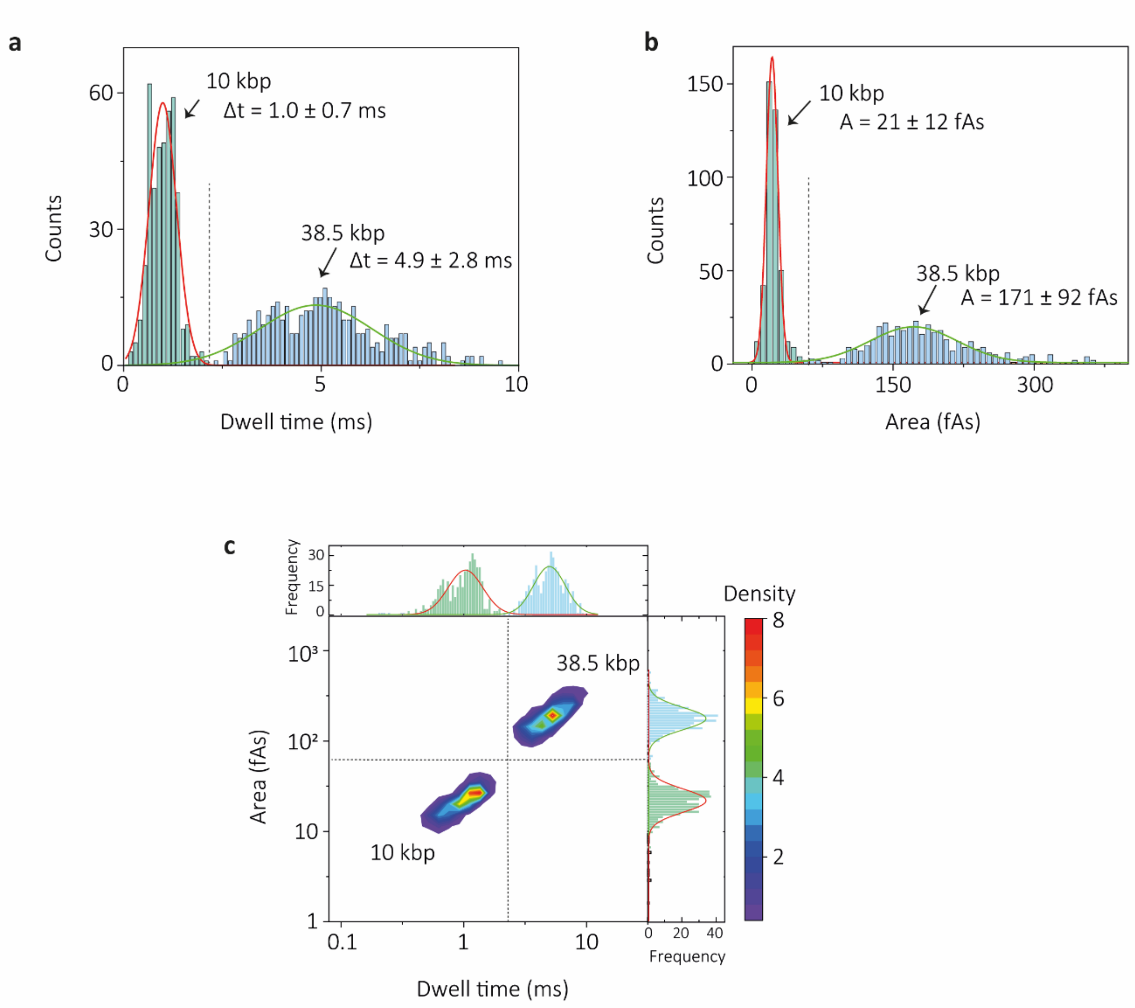

Supplementary Fig. 7 | 10 and 38.5 kbp DNA carrier translocation statistics

Histograms for dwell time **(a)** and peak current area (the integrated current over event dwell time) **(b)** for nanopore detection of 10 and 38.5 kbp DNA. Two distinct current distributions were observed, one with a shorter dwell time (Δt = 1.0 ± 0.7 ms, mean ± s.d.) and smaller peak current area (A = 21 ± 12 fAs, mean ± s.d.) and another with a longer dwell time (Δt = 4.9 ± 2.8 ms) and larger peak current area (A = 171 ± 92 fAs). Distributions were fitted with Gaussian functions. **(c)** Density scatter plots of the integrated current area vs dwell time showing two distinctive population. All the measurements were performed in 100 mM KCl buffer (5 mM $MgCl_2$, 10 mM Tris-HCl, 1 mM EDTA, pH = 8.0).

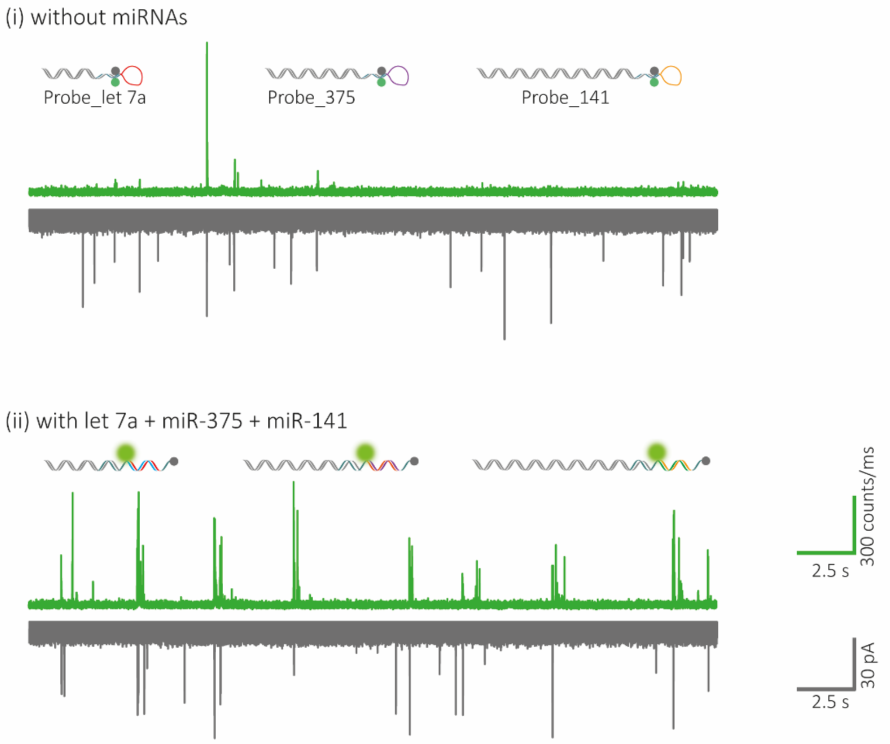

Supplementary Fig. 8 | Simultaneous detection of three miRNA sequences.

Representative photon and current-time traces for the translocation of Probe-let 7a (5.6 kbp), Probe-375 (10 kbp) and Probe-141 (38.5 kbp) (10 pM per probe) at the absence (i) and presence (ii) of let 7a, miR-375, and miR-141 (all probes are at concentration of 10 pM). All the translocations were performed at -300 mV in 100 mM KCl buffer (5 mM $MgCl_2$, 10 mM Tris-HCl, 1 mM EDTA, pH = 8.0). Laser power is 90 ± 4 µW.

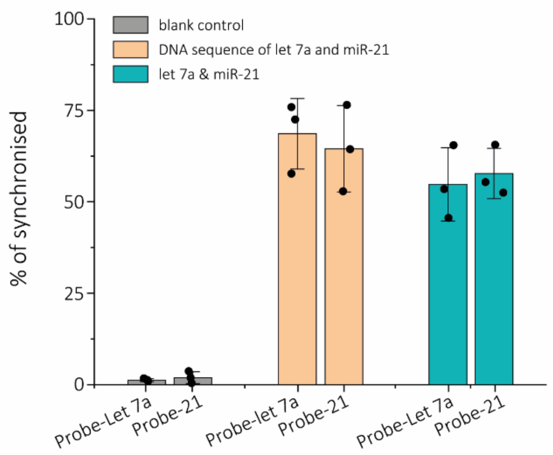

Supplementary Fig. 9 | Single-molecule electro-optical detection of let 7a and miR-21 and the DNA analogues.

Synchronisation ratio of nanopore translocated Probe-let 7a (10 kb) and Probe-21 (38.5 kb), on their own (grey), and with the complementary DNA oligos (beige), and in combination with let 7a and miR-21 miRNAs (teal). All the molecular probes and targets have a concentration of 10 pM. All translocation experiments were performed at -300 mV in 100 mM KCl buffer (5 mM $MgCl_2$, 10 mM Tris-HCl, 1 mM EDTA, pH = 8.0). The laser power was 90 ± 4 µW. All error bars represent the standard deviation for data measured from 3 different nanopipettes (n = 3). Data are presented as mean ± s.d.

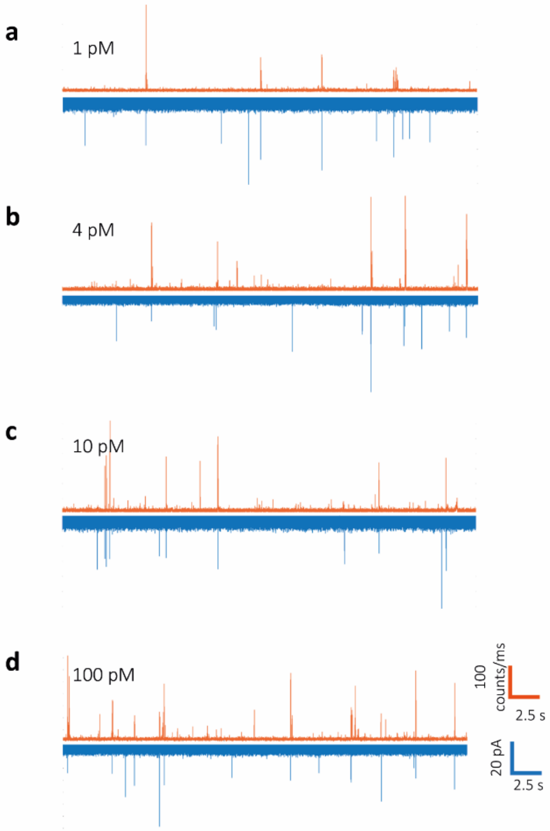

Supplementary Fig. 10 | Photon and Current-time traces for concentration dependence.

(a-d) Photon and current-time traces for the Probe-375 (10 kbp) and Probe-141 (38.5 kbp) translocations with increasing concentration of miR-375 and miR-141 (from 1 pM to 100 pM). All the translocations were performed at -300 mV in 100 mM KCl buffer (5 mM $MgCl_2$, 10 mM Tris-HCl, 1 mM EDTA, pH = 8.0). The laser power was 90 ± 4 µW.

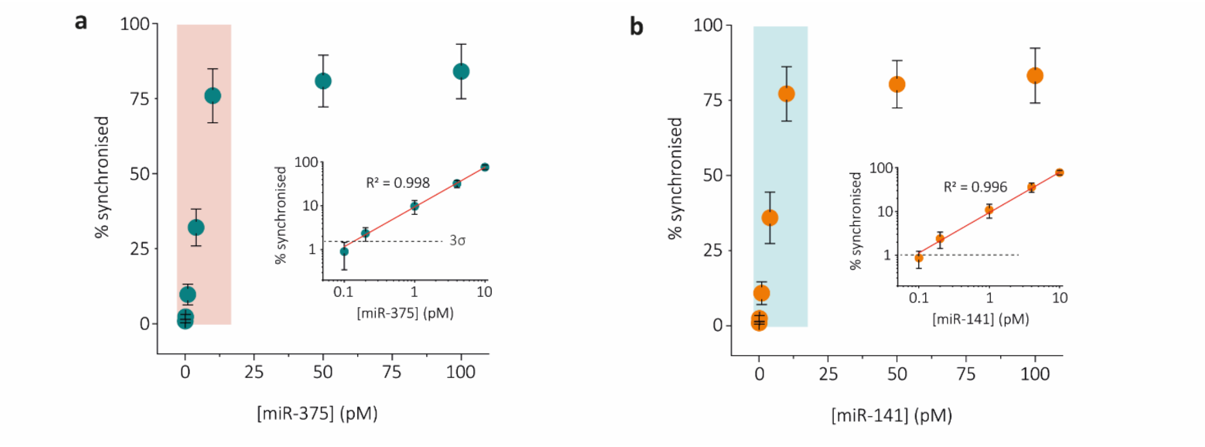

Supplementary Fig. 11 | Calibration curves for concentration dependence at symmetric salt solution.

(**a-b**) Calibration curves of synchronisation ratio at different concentrations of miR-375 (**a**) and miR-141 (**b**) in symmetric salt buffer conditions (cis & trans: 100 mM KCl, 5 mM MgCl2, 10 mM Tris-HCl, 1 mM EDTA, pH = 8.0). In all cases, the concentrations of molecular probes are kept at 10 pM. Both the insets show a linear increase of % synchronised within the miRNA concentration range of 0.1 to 10 pM. The correlation coefficients (R^2 value) are 0.998 and 0.996 for miR-375 and miR-141, respectively. The dashed lines represent 3-times the standard deviation (3σ) of % synchronised events for blank controls. All the measurements were performed with 3 independent nanopores (n = 3). Data are presented as mean ± s.d.

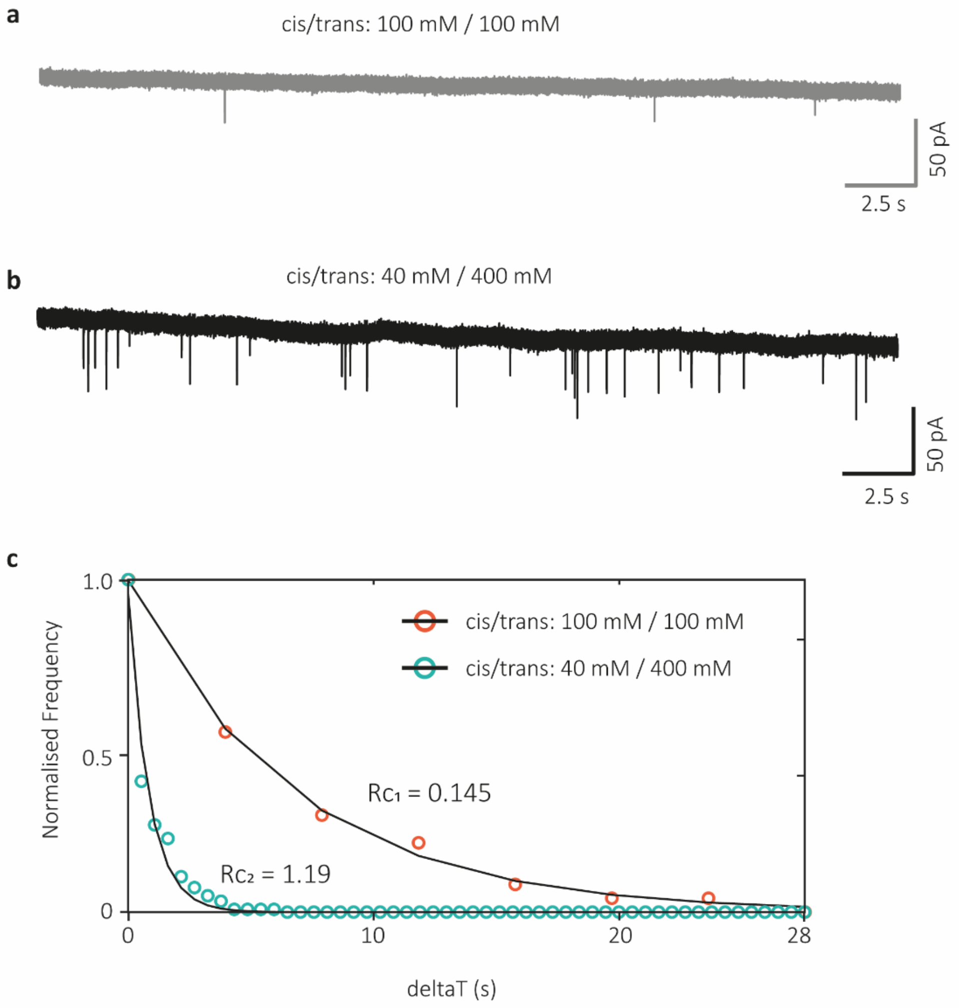

Supplementary Fig. 12 | Capture rate enhancement using a salt gradient

Current-time traces for the translocation of 10 kbp and 38.5 kbp DNA probes through a nanopipette at **(a)** symmetric 100 mM KCl and **(b)** asymmetric 40 mM (inside nanopore) and 400 mM KCl (outside nanopore) concentration. **(c)** Normalised distributions of the time elapsed between successive events (δt) in symmetric and asymmetric salt concentrations using a bias of -300 mV. Solid lines represent a single-exponential decay fit, from which the DNA capture rate (R_c) is extracted.

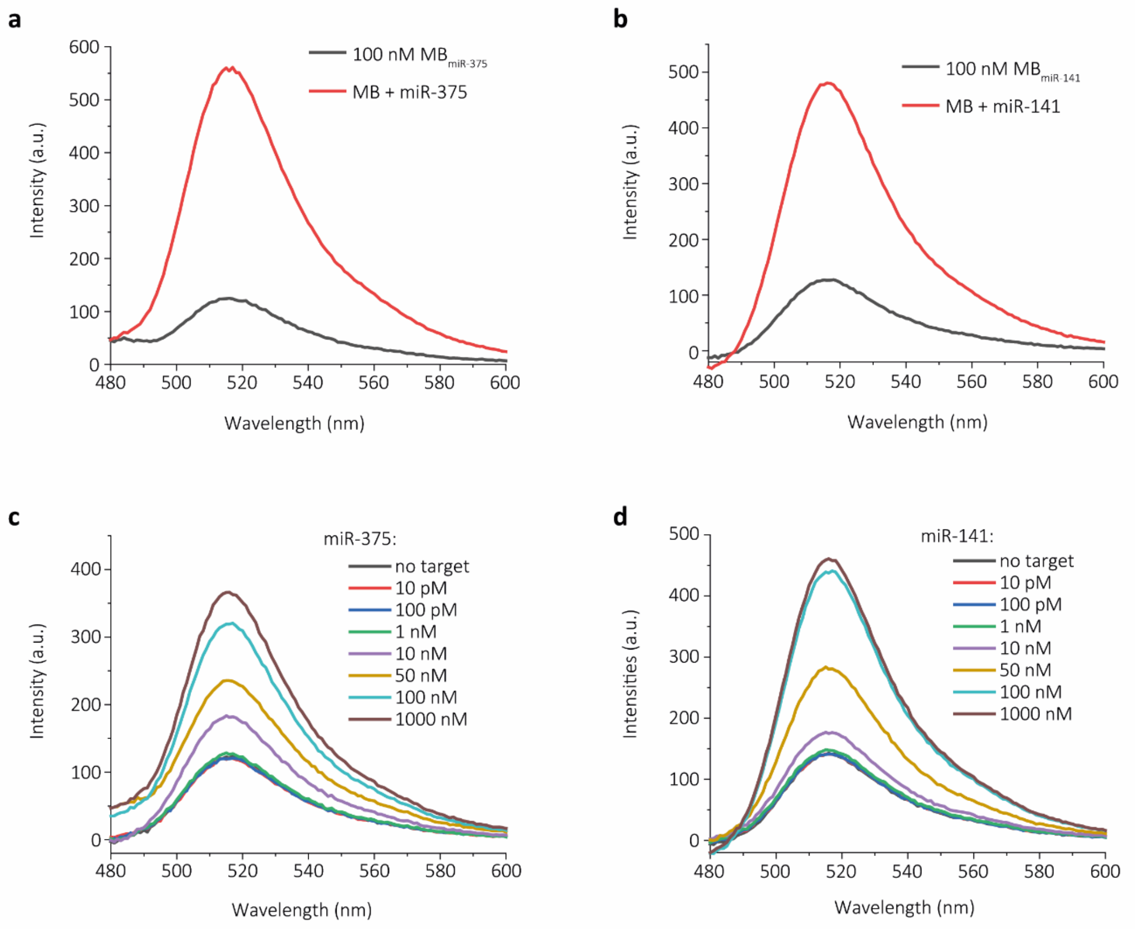

Supplementary Fig. 13 | Bulk fluorescence measurement.

Fluorescence spectrum of 100 nM MB_{miR375} **(a)** and MB_{miR141} **(b)** in the absence (black curve) and presence (red curve) of 10× excess of target miRNAs. Fluorescence spectrum of 100 nM MB_{miR375} **(c)** and MB_{miR141} **(d)** with an increasing concentration of target miRNAs from 10 pM to 1 µM. All MBs were hybridised with short cDNA oligos (BTA1 or BTA2, sequences can be found in Supplementary Table 1) in excess of 10×. The cDNA oligos had sequences, complementary to the tail of the MBs, in order to mimic the attachment to the 12-base sticky overhang of the DNA carrier. All fluorescence was measured in the 100 mM KCl buffer (5 mM $MgCl_2$, 10 mM Tris-HCl, 1 mM EDTA, pH = 8.0) with 460 nm excitation. a.u., arbitrary units.

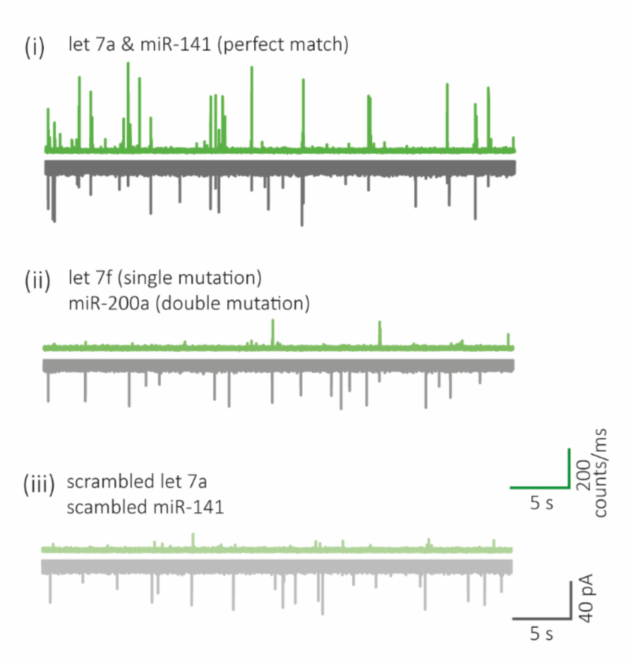

Supplementary Fig. 14 | Photon and current time traces for single-/double-base mutation selectivity.

Photon and current time traces are shown for the translocation of Probe-let 7a (10 pM), and Probe-141 (10 pM) in the presence of (i) let-7a and miR-141, (ii) let-7f and miR-200a, and (iii) scrambled sequences. All miRNAs were added at 10 pM. These experiments were performed under symmetric KCl salt conditions (100 mM with 5 mM $MgCl_2$, 10 mM Tris-HCl, 1 mM EDTA, pH = 8.0) at -300 mV. The laser power used was 90 ± 4 µW for all the measurements.

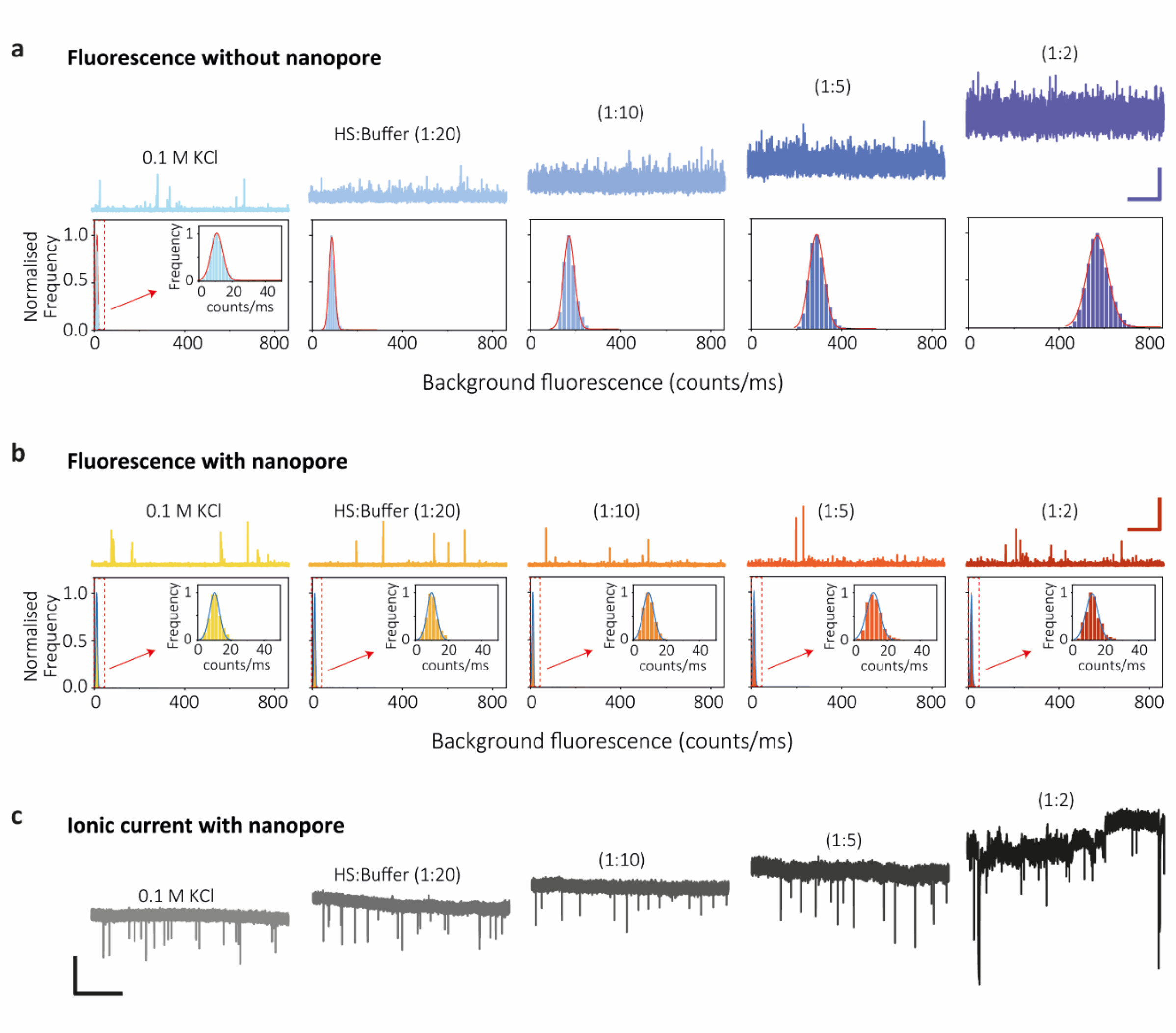

Supplementary Fig. 15 | Measurements in a physiologically compatible buffer and in human serum.

(a) Photon-time traces for detection of miR-375 and miR-141 using a conventional single-molecule confocal method in a physiologically compatible buffer (0.1 M KCl, 5 mM MgCl2, 10 mM Tris-HCl, 1 mM EDTA, pH = 8.0) and in a human serum diluted in the same buffer (serial dilution from 1:20 to 1:2). Scale bars, vertical: 200 counts/ms, horizontal: 2s. **(b)** Traces to those in recorded **(a)** using the electro-optical sensing platform with nanopore. Scale bar, vertical: 200 counts/ms, horizontal: 2s. The histograms underneath the traces showed the background photon counts. While the background showed a significant increase with the increasing concentration of human serum in the buffer, the electro-optical platform shows identical background for all concentrations. All histograms were fitted with Gaussian function. **(c)** Current-time traces for nanopore translocations of Probe-375 (10 kb) and Probe-141 (38.5 kb) and in a human serum diluted in the same buffer. Scale bar, vertical: 50 pA, horizontal: 5s.

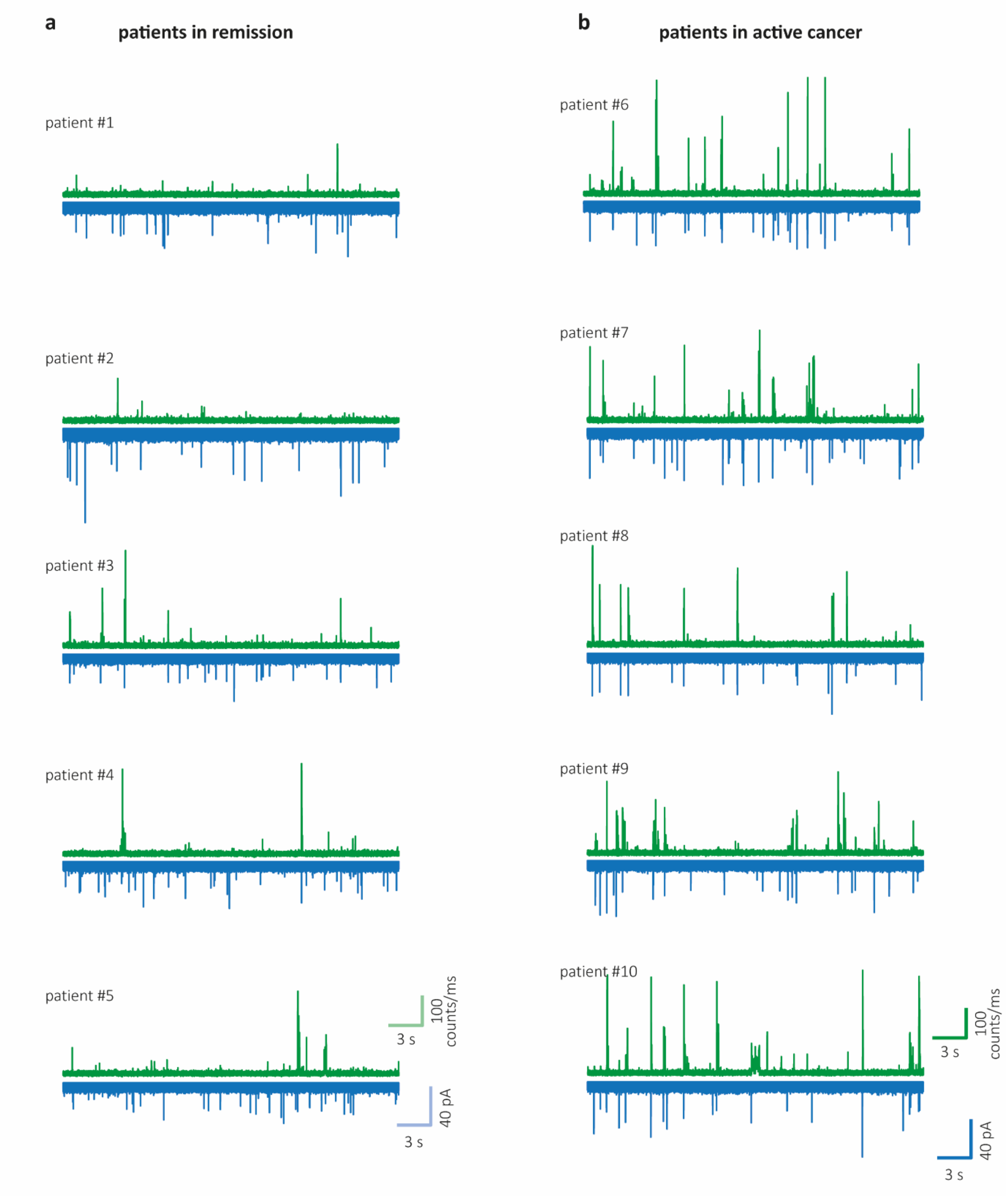

Supplementary Fig. 16 | miR-375 and miR-141 detection directly from prostate cancer serum.

Representative photon and current-time traces for the detection of miR-375 and miR-141 directly from serum for 5 patients in remission **(a)** and 5 patients with active prostate cancer **(b)**. All the translocations were performed at -300 mV in an asymmetric KCl buffer (40 mM inside and 400 mM outside nanopipette). The laser power was 90 ± 4 µW.

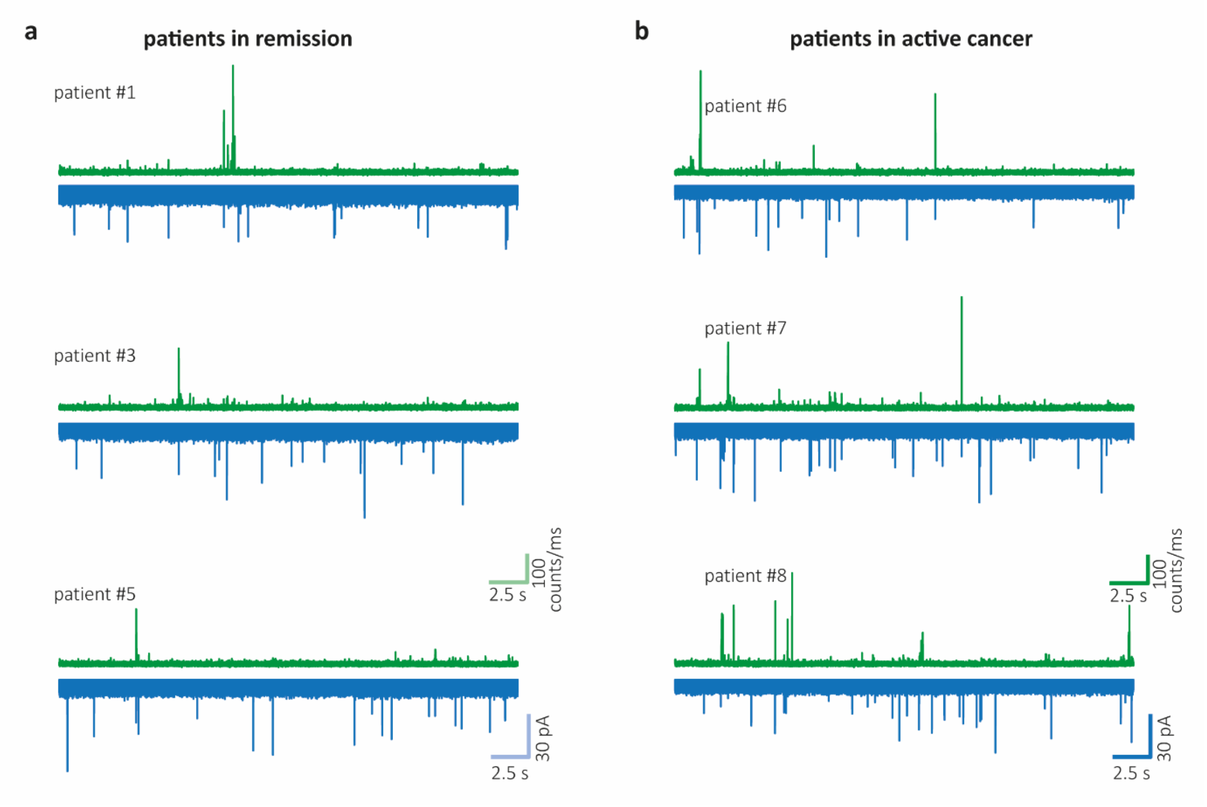

Supplementary Fig. 17 | miR-375 and miR-141 detection from serum extracts.

Representative photon and current-time traces for the detection of miR-375 and miR-141 from 3 patients in remission **(a)** and 3 patients with active prostate cancer **(b)**. All the translocations were performed at -300 mV in 100 mM KCl buffer (5 mM $MgCl_2$, 10 mM Tris-HCl, 1 mM EDTA, pH = 8.0). The laser power was 90 ± 4 µW.

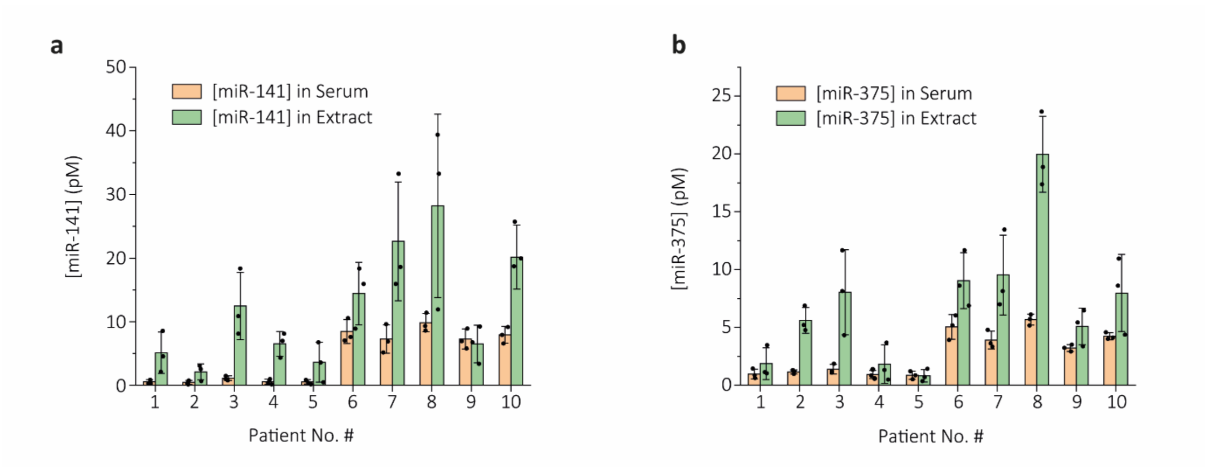

Supplementary Fig. 18 | Comparison of miR-141 and miR-375 expression level detected in miRNA extracts and in serum.

Concentrations of miR-141 **(a)** and miR-375 **(b)** as calculated from the calibration curves presented in Fig. 4 in the main text and Supplementary Fig. 11. All error bars represent the standard deviation for data measured from 3 different nanopipettes (n = 3). Data are presented as mean ± s.d.

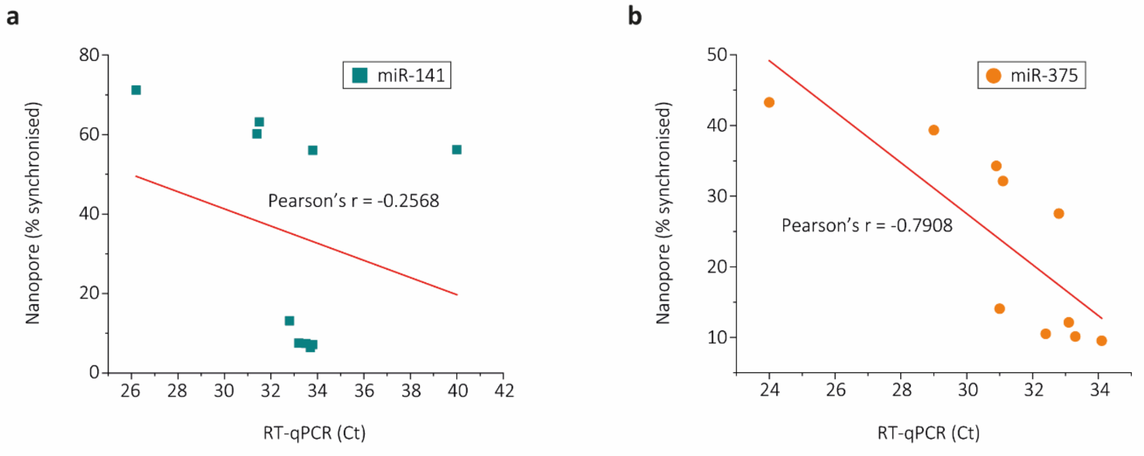

Supplementary Fig. 19 | Correlation of miRNA expression levels detected using RT-qPCR and nanopores directly in human serum.

Correlation between % synchronised measured directly in serum and Ct value measured using RT-qPCR for miR-141 **(a)** and miR-375 **(b)** respectively. Pearson's r for miR-141, r = -0.2568, P = 0.4735, and for miR-375, r = -0.7908, P = 0.0064. Statistical significance was tested using two-tailed Student's t-test.

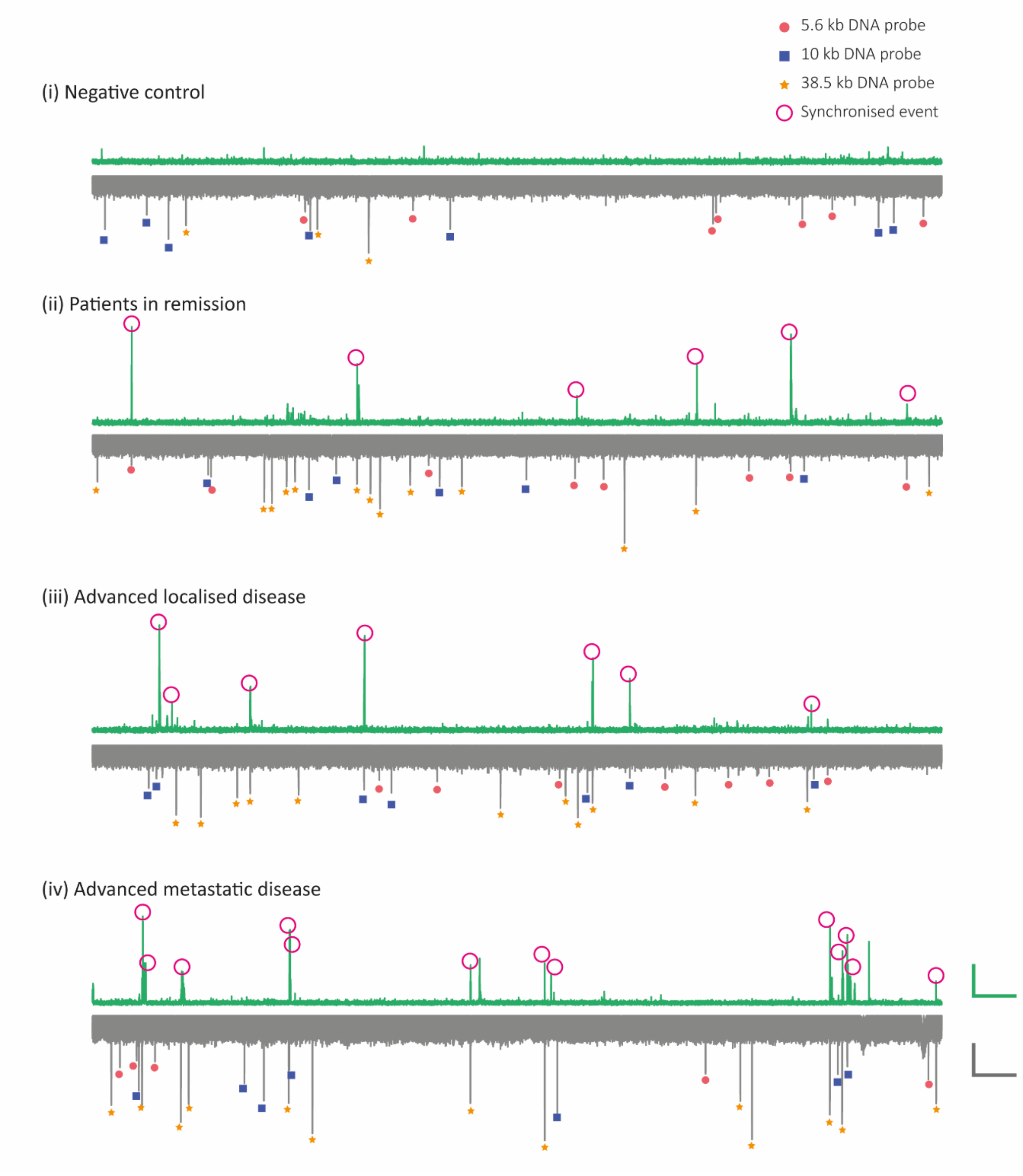

Supplementary Fig. 20 | 3 miRNAs profiling in prostate cancer patients.

Photon and current time traces are shown for the translocation of Probe-let 7b (5.6 kb), Probe-375 (10 kb), and Probe-141 (38.5 kb) in the (i) absence and presence of patient serum with stages of (ii) remission, (iii) advanced localised, and (iv) advanced metastatic diseases. Electrical signals representing the translocation events of 5.6, 10 and 38.5 kbp DNA fragment lengths are marked with a filled circle, square and asterisk. Signals in the optical channel with corresponding synchronised events are marked with circles. All DNA molecular probes were added at 1 pM. These experiments were performed at -300 mV in an asymmetric KCl buffer (40 mM inside and 400 mM outside nanopipette). The laser power was 90 ± 4 µW. Scale bar of photon trace (green): vertical, 100 counts/ms, horizontal, 2.5 s. Scale bar of current trace: vertical, 20 pA, horizontal, 2.5 s.

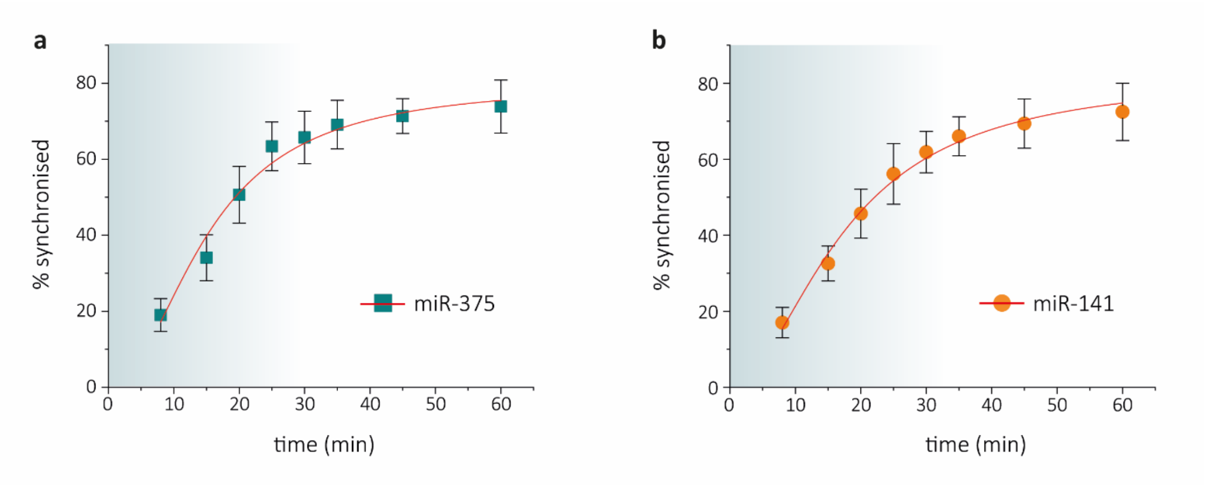

Supplementary Fig. 21 | Binding dynamics.

Time-dependence on the synchronisation ratio events for miR-375 **(a)** and miR-141 **(b)**. All error bars represent the standard deviation for data measured from 3 different nanopipettes (n = 3). Data are presented as mean ± s.d. All the measurements were performed at a bias of -300 mV in 100 mM KCl buffer (5 mM $MgCl_2$, 10 mM Tris-HCl, 1 mM EDTA, pH = 8.0). The laser power was 90 ± 4 µW.

Supplementary Note 1. Estimation of hybridisation kinetics (k_h)

The half-file of the reaction of MB hybridisation with its target miRNA sequences can be expressed by the following equation (1), where $t_{1/2}$ is the half-life, k_h is the hybridisation rate constant, and [A] represents the concentration of substate. By fitting the binding kinetic curves in Supplementary Fig. 21, we obtained half-life of 15 min and 17 min, respectively. Assuming the concentration of MB is 10 pM, we calculated the rate constant for MB-375 and MB-141 are 7.7×10^7 $M^{-1}s^{-1}$ and 6.8×10^7 $M^{-1}s^{-1}$, respectively. These values are approximately 2 to 3 orders of magnitude larger than the literature reported[3]. However, as reported by previously reported works[4, 5], the nanopipette can concentrated the charged analytes near the tip due to the electroosmotic flow up to ~1,000 folds. Taking this into account, the realistic rate constant (k_h) should be much smaller than those estimated above.

$$t_{1/2} = \ln 2/(k_h \times [A]) \qquad (1)$$

Supplementary Tables

Supplementary Table 1. Sequences of DNA and miRNAs used

Oligos	Sequences	Specification
MB_(miR-375)	5'-*AGGTCGCCGCCC* T(**Alexa 488**) CCGTACG T CAC GCG AGC CGA ACG AAC AAA CGTACGGA-**Dabcyl**-3'	The bases in italics indicate the complementary part to the sticky overhang of λ-DNA. The underlined bases form the stem of the hairpin structure.
MB_(miR-141)	5'-*GGGCGGCGACCT* T(**Alexa 488**) CCGGCAC C CAT CTT TAC CAG ACA GTG TTA GTGCCGGA-**Dabcyl**-3'	
MB_(let 7a)	5'-*AGGTCGCCGCCC* T(**Alexa 488**) CTGCAAC AAC TAT ACA ACC TAC TAC CTC A GTTGCAGA-**Dabcyl**-3'	
MB_(miR-21)	5'-*GGGCGGCGACCT* T(**Alexa 488**) CTTGGAC TCA ACA TCA GTC TGA TAA GCTA GTCCAAGA-**Dabcyl**-3'	
MB_(let 7b)	5'- *AGGTCGCCGCCC* T(**Alexa 488**) CCGTGCG A ACC ACA CAA CCT ACT ACC TCA CGCACGGA-**Dabcyl**-3'	
let 7a_DNA	5'-TGA GGT AGT AGG TTG TAT AGT T-3'	DNA analogue of let 7a
miR-21_DNA	5'-TAG CTT ATC AGA CTG ATG TTG A-3'	DNA analogue of miR-21
let 7a	5'-UGA GGU AGU AGG UUG UAU AGU U-3'	
miR-21	5'-UAG CUU AUC AGA CUG AUG UUG A-3'	
miR-375	5'-UUU GUU CGU UCG GCU CGC GUG A-3'	PCa biomarkers (Ref.[6-8])
miR-141	5'-UAA CAC UGU CUG GUA AAG AUG G-3'	
let 7b	5'-UGA GGU AGU AGG UUG UGU GGU U-3'	
let 7f	5'-UGA GGU AGU AGA UUG UGU GGU U-3'	Single-mismatched with Let 7a
miR-200a	5'-UAA CAC UGU CUG GUA ACG AUG U-3'	Same family with miR-141 with double-mismatched
Scrambled let 7a	5'-GUU AUG UUG GUU AGU GGA UUAA-3'	Scrambled sequence for let 7a
Scrambled miR-141	5'-AUG AGU GAG AGA UAC GCU UCU A -3'	Scrambled sequence for miR-141
BTA1	5'-GGG CGG CGA CCT TTT-Biotin-3'	complementary to the tail of MB
BTA2	5'-AGG TCG CCG CCC TTT-Biotin-3'	

MB = molecular beacon

Supplementary Table 2. Comparison of time and sample size required for current methods and nanopore sensing

Methods	Steps and time required	Total time	Sample volume required	Reference
RT-qPCR	1. Serum preparation, including blood clotting and centrifuging. (40 min)<br>2. RNA extraction. (1.5-3 hours)<br>3. Reverse transcription. (2-3 hours)<br>4. Real-time quantitative PCR. (1.5-3 hours)	6-10 hours	≥200 µl	Ref. [9, 10]
Microarray	1. Serum preparation. (40-70 min)<br>2. RNA extraction. (1-2 hours)<br>3. miRNA target preparation and labelling. (2-3 hours)<br>4. miRNA array hybridisation, signal detection and array scanning. (24 hours)	~28-32 hours	>1 ml	Ref. [11, 12]
RNA-Seq	1. RNA extraction. (2-3 hours)<br>2. Ligation of 3' and 5' adapters. (18 hours)<br>3. Reverse transcription. (2 hours)<br>4. PCR amplification and purification. (2-3 hours)<br>5. Quality control and size selection. (3 hours)<br>6. Sequencing and data processing. (≥2 days)	>3 days	>1 ml	Ref. [13]
Nanopore (this work)	1. Serum preparation, including blood clotting and centrifuging. (40 min)<br>2. Incubation probes with serum. (30-35 min)<br>3. Nanopore electro-optical assay. (10 min)	<1.5 hours	0.1 µl	This work

Supplementary References

1. Cai SL, Sze JYY, Ivanov AP, Edel JB. Small molecule electro-optical binding assay using nanopores. *Nat Commun* **10**, 9 (2019).

2. Pitchford WH*, et al.* Synchronized Optical and Electronic Detection of Biomolecules Using a Low Noise Nanopore Platform. *ACS Nano* **9**, 1740-1748 (2015).

3. Kuhn H, Demidov VV, Coull JM, Fiandaca MJ, Gildea BD, Frank-Kamenetskii MD. Hybridization of DNA and PNA molecular beacons to single-stranded and double-stranded DNA targets. *J Am Chem Soc* **124**, 1097-1103 (2002).

4. Chang PL, Graf M, Hung CH, Radenovic A. Orthogonal Tip-to-Tip Nanocapillary Alignment Allows for Easy Detection of Fluorescent Emitters in Femtomolar Concentrations. *Nano Lett* **18**, 3165-3171 (2018).

5. Zhang DW*, et al.* Microfabrication-free fused silica nanofluidic interface for on chip electrokinetic stacking of DNA. *Microfluid Nanofluid* **14**, 69-76 (2013).

6. Porzycki P, Ciszkowicz E, Semik M, Tyrka M. Combination of three miRNA (miR-141, miR-21, and miR-375) as potential diagnostic tool for prostate cancer recognition. *Int Urol Nephrol* **50**, 1619-1626 (2018).

7. Mitchell PS*, et al.* Circulating microRNAs as stable blood-based markers for cancer detection. *Proc Natl Acad Sci U S A* **105**, 10513-10518 (2008).

8. Zedan AH, Hansen TF, Assenholt J, Pleckaitis M, Madsen JS, Osther PJS. microRNA expression in tumour tissue and plasma in patients with newly diagnosed metastatic prostate cancer. *Tumour Biol* **40**, 1010428318775864 (2018).

9. Schmittgen TD, Zakrajsek BA, Mills AG, Gorn V, Singer MJ, Reed MW. Quantitative reverse transcription-polymerase chain reaction to study mRNA decay: Comparison of endpoint and real-time methods. *Anal Biochem* **285**, 194-204 (2000).

10. Schmittgen TD, Jiang JM, Liu Q, Yang LQ. A high-throughput method to monitor the expression of microRNA precursors. *Nucleic Acids Res* **32**, 10 (2004).

11. Liu CG*, et al.* An oligonucleotide microchip for genome-wide microRNA profiling in human and mouse tissues. *Proc Natl Acad Sci U S A* **101**, 9740-9744 (2004).

12. Liu CG, Calin GA, Volinia S, Croce CM. MicroRNA expression profiling using microarrays. *Nat Protoc* **3**, 563-578 (2008).

13. Hagemann-Jensen M, Abdullayev I, Sandberg R, Faridani OR. Small-seq for single-cell small-RNA sequencing. *Nat Protoc* **13**, 2407-2424 (2018).
