# Supplementary material for: Single-molecule amplification-free multiplexed detection of circulating microRNA cancer biomarkers from serum
Source: Nat Commun. 2021 Jun 10;12:3515. doi: 10.1038/s41467-021-23497-y (PMC8192752; doi:10.1038/s41467-021-23497-y)
Supplement: Supplementary file 2 — Reporting Summary [file 41467_2021_23497_MOESM2_ESM.pdf]

nature research

Corresponding author(s): Joshua B. Edel

Last updated by author(s): Apr 15, 2021

Reporting Summary

Nature Research wishes to improve the reproducibility of the work that we publish. This form provides structure for consistency and transparency in reporting. For further information on Nature Research policies, see our Editorial Policies and the Editorial Policy Checklist.

Statistics

For all statistical analyses, confirm that the following items are present in the figure legend, table legend, main text, or Methods section.

n/a	Confirmed	
☐	☒	The exact sample size (n) for each experimental group/condition, given as a discrete number and unit of measurement
☐	☒	A statement on whether measurements were taken from distinct samples or whether the same sample was measured repeatedly
☐	☒	The statistical test(s) used AND whether they are one- or two-sided<br>*Only common tests should be described solely by name; describe more complex techniques in the Methods section.*
☒	☐	A description of all covariates tested
☒	☐	A description of any assumptions or corrections, such as tests of normality and adjustment for multiple comparisons
☐	☒	A full description of the statistical parameters including central tendency (e.g. means) or other basic estimates (e.g. regression coefficient) AND variation (e.g. standard deviation) or associated estimates of uncertainty (e.g. confidence intervals)
☐	☒	For null hypothesis testing, the test statistic (e.g. F, t, r) with confidence intervals, effect sizes, degrees of freedom and P value noted<br>*Give P values as exact values whenever suitable.*
☒	☐	For Bayesian analysis, information on the choice of priors and Markov chain Monte Carlo settings
☒	☐	For hierarchical and complex designs, identification of the appropriate level for tests and full reporting of outcomes
☐	☒	Estimates of effect sizes (e.g. Cohen's d, Pearson's r), indicating how they were calculated

Our web collection on statistics for biologists contains articles on many of the points above.

Software and code

Policy information about availability of computer code

Data collection	A NI 6602 DAQ card (National Instruments) was coupled with the APDs for obtaining the optical data while another NI-USB 6251 DAQ card (National Instruments) was used for the electrical data collection. The electrical data were collected using a software of WinWCP (V5.4.2) and optical data were collected using Labview (2014 SP1, 64-bit) software. The synchronisation of electrical and optical detection was achieved through a connection between these two cards and triggered via the application of a TTL pulse using a custom-written Labview (2014 SP1, 64-bit) program.
Data analysis	All the electrical signals were sampled at 100 KHz kHz and filtered at 10 KHz kHz using a low-pass Bessel filter. Optical photon counts were collected via APD detectors with a time resolution of 10 µs. A custom-written application in Matlab (R2019b) was used to analyse synchronised optical-electrical events. Electrical and optical data were initially analysed independently using this application. The obtained optical and electrical events were then further analysed using a Coincident App based on Matlab (R2019b) and Origin 2019. Plots and charts were performed using Origin 2019. Adobe Illustrator (2020) was used to draw schematics.

For manuscripts utilizing custom algorithms or software that are central to the research but not yet described in published literature, software must be made available to editors and reviewers. We strongly encourage code deposition in a community repository (e.g. GitHub). See the Nature Research guidelines for submitting code & software for further information.

Data

Policy information about availability of data

All manuscripts must include a data availability statement. This statement should provide the following information, where applicable:

- Accession codes, unique identifiers, or web links for publicly available datasets
- A list of figures that have associated raw data
- A description of any restrictions on data availability

All data support the findings are presented within this paper and its Supplementary Information. Additional data that support the findings of this study are available from the corresponding author upon reasonable request.

Field-specific reporting

Please select the one below that is the best fit for your research. If you are not sure, read the appropriate sections before making your selection.

☒ Life sciences ☐ Behavioural & social sciences ☐ Ecological, evolutionary & environmental sciences

For a reference copy of the document with all sections, see nature.com/documents/nr-reporting-summary-flat.pdf

Life sciences study design

All studies must disclose on these points even when the disclosure is negative.

Sample size	The clinical sample analysis, at least 5 patient samples were tested for remission or active cohort. No statistical methods were used to determine sample size. The sample size were decided according to a previous study using the same cohorts of samples.
Data exclusions	No data were excluded.
Replication	Values described in the biological samples were obtained from 3 technical replicates. All attempts of replication were successful.
Randomization	Patient samples were allocated into different cohorts (remission, active etc.) according to the PSA results before testing.
Blinding	Data collection and analysis were not blinded. Blinding was not relevant because the main purpose of the study was to test the performance of the Electro-optical nanopore system. The samples tested has been reported in previous study.

Reporting for specific materials, systems and methods

We require information from authors about some types of materials, experimental systems and methods used in many studies. Here, indicate whether each material, system or method listed is relevant to your study. If you are not sure if a list item applies to your research, read the appropriate section before selecting a response.

Materials & experimental systems

n/a	Involved in the study	
☒	☐	Antibodies
☒	☐	Eukaryotic cell lines
☒	☐	Palaeontology and archaeology
☒	☐	Animals and other organisms
☐	☒	Human research participants
☒	☐	Clinical data
☒	☐	Dual use research of concern

Methods

n/a	Involved in the study	
☒	☐	ChIP-seq
☒	☐	Flow cytometry
☒	☐	MRI-based neuroimaging

Human research participants

Policy information about studies involving human research participants

Population characteristics	Whole blood from patients who attended clinics at Imperial College Healthcare NHS Trust (London, UK) with different stages of prostate cancer was collected in 2014 following written patient consent. Age, gender of participants are random. The age and gender are not relevant to this study.
Recruitment	Patients were volunteered to participate the research following written patient consent. Whole blood samples were obtained with ethical approval from patients attending clinics at Imperial College London NHS Healthcare Trust (London, UK). All samples were taken from patients in 2014 following written patient consent and stored as a subcollection in the Imperial College Healthcare Trust Tissue Bank.

Ethics oversight	This study that relevant to human participants has been approved by Wales Research Ethics Committees (RECs).

Note that full information on the approval of the study protocol must also be provided in the manuscript.
